# Supplementary material for: A shape-shifting nuclease unravels structured RNA
Source: Nat Struct Mol Biol. 2023 Feb 23;30(3):339–47. doi: 10.1038/s41594-023-00923-x (PMC10023572; doi:10.1038/s41594-023-00923-x)

nature structural & molecular biology

Article
https://doi.org/10.1038/s41594-023-00923-x

A shape-shifting nuclease unravels structured RNA

In the format provided by the authors and unedited

Supplementary Information

A shape-shifting nuclease unravels structured RNA

Katarina Meze[1,2,3], Armend Axhemi[1,3], Dennis R. Thomas[1,3], Ahmet Doymaz[2,4]

and Leemor Joshua-Tor[1,2,3*]

[1]W.M. Keck Structural Biology Laboratory, Howard Hughes Medical Institute, 1 Bungtown Road, Cold Spring Harbor, 11724, USA.

[2]Cold Spring Harbor School of Biological Sciences

[3]Cold Spring Harbor Laboratory, 1 Bungtown Road, Cold Spring Harbor, NY, 11724, USA.

[4]Current: Weill Cornell/Rockefeller/Sloan Kettering Tri-Institutional MD-PhD Program, New York, NY 10065, USA

*To whom correspondence should be addressed

Correspondence should be addressed to L.J. leemor@cshl.edu

Supplementary Information Guide

Supplementary Video 1. Figure Legend 3

Data Exclusion Information 3

Supplementary Tables

Supplementary Table 1. Asymmetric error analysis for WT Dis3L2 on hairpinA-GCU$_{14}$ 4

Supplementary Table 2. Asymmetric error analysis for ΔCSD on hairpinA-GCU$_{14}$ 4

Supplementary Table 3. Asymmetric error analysis for Δ123H on hairpinA-GCU$_{14}$ 4

Supplementary Table 4. Sample size and replicates for kinetic studies of WT and mutant Dis3L2 with different substrates 5

Supplementary Figures

Supplementary Figure 1. Representative cryoEM data processing pathways 6

Supplementary Figure 2. RNA-free HsDis3L2^{D391N} 8

Supplementary Figure 3. RNA-free WT HsDis3L2 9

Supplementary Figure 4. WT HsDis3L2^{D391N} HairpinA-GCU$_{14}$ 10

Supplementary Figure 5. WT HsDis3L2 HairpinC-U$_{12}$ 11

Supplementary Figure 6. WT HsDis3L2 HairpinD-U$_9$ 12

Supplementary Figure 7. WT HsDis3L2 HairpinD-U$_9$ #2 13

Supplementary Figure 8. WT HsDis3L2 HairpinD-U$_8$ 14

Supplementary Figure 9. WT HsDis3L2 HairpinD-U$_8$ #2 15

Supplementary Figure 10. WT HsDis3L2 HairpinD-U$_9$ +EDTA 16

Supplementary Figure 11. WT HsDis3L2 HairpinD-U$_7$ 17

Supplementary Figure 12. WT HsDis3L2 HairpinD-U$_7$ #2 18

Supplementary Figure 13. WT HsDis3L2 HairpinD-U$_5$ 19

Supplementary Figure 14. WT HsDis3L2 HairpinD-U$_5$ +EDTA 20

Supplementary Video 1. Structural changes during the different stages of structured RNA degradation by Dis3L2. The RNA-free Dis3L2 and domain composition in the vase conformation are shown, followed by the complex of Dis3L2 with hairpinA-GCU$_{14}$. The latter represents initial substrate binding at which point the dsRNA is not engaged by the enzyme. After shortening of the overhang to roughly 12 nt, the double-helix is cradled between the S1 and CSD domains, shown here by the structure of the complex of Dis3L2 with hairpin-U$_{12}$. When the overhang length is roughly 8 nt long, the structured portion of the RNA substrate would clash with the enzyme and triggers a dramatic 70 Å domain movement of CSDs to the other side of the enzyme. A morph created in ChimeraX shows the transition between the vase and prong conformation, with the side view of the transition highlighting the large distance that the CSDs travel. This conformational change exposes the trihelix-linker that acts as a wedge to separate the two RNA strand and shortens the approach to the active site. The double-helix is further stabilized by the S1 domain in the structure of the complex of Dis3L2 with hairpinD-U$_7$.

Data Exclusion Information

Data exclusion in cryoEM: CryoEM data sample size was defined by the number of particles picked using WarpEM's software. Particles with a minimum of 40Å distance from contamination or grid bars, below 4Å estimated resolution, with local defocus value of between 0.4 and 3 micro-meters, and up to 1.5Å motion per frame were collected. 2D classification was used to select for the best particles for *ab initio* reconstructions (criteria are outlined in the methods). Particles were excluded by 3D classification (heterogeneous refinement in CryoSPARC), and only particles comprising the highest resolution classes selected for further processing.

Datapoints excluded in the final kinetics graphs:

Datapoints that were excluded from the graph are reported in the figure legends. List of excluded datapoints due to large uncertainty:

Figure 3. WT Dis3L2 association rate data point x=-8.

Extended Data Fig. 7. WT Dis3L2 non-productive binding data point for x=15.

Extended Data Fig. 7. WT Dis3L2 processivity on HairpinB-GCU14, datapoint x=-11.

Extended Data Fig. 9. ΔCSD association rate data point for x=-3 and -4.

Supplementary Table 1. Asymmetric error analysis for WT Dis3L2 on hairpinA-GCU$_{14}$

Parameter	Units	Best Fit	Upper Bound	Lower Bound
k_{on}^{P}	$nM^{-1}\ min^{-1}$	0.180	0.200	0.159
k_{off}^{P}	min^{-1}	92.0	107	86
$k_{forward}$	min^{-1}	28.5	32.0	25.5
k_{off}^{NP}	min^{-1}	0.125	0.152	0.0770

Supplementary Table 2. Asymmetric error analysis for ΔCSD on hairpinA-GCU$_{14}$

Parameter	Units	Best Fit	Upper Bound	Lower Bound
k_{on}^{P}	$nM^{-1}\ min^{-1}$	0.258	0.296	0.225
k_{off}^{P}	min^{-1}	25.2	28.9	21.9
$k_{forward}$	min^{-1}	2.44	2.74	2.15
k_{off}^{NP}	min^{-1}	0.0805	0.129	0.0338

Supplementary Table 3. Asymmetric error analysis for Δ123H on hairpinA-GCU$_{14}$

Parameter	Units	Best Fit	Upper Bound	Lower Bound
k_{on}^{P}	$nM^{-1}\ min^{-1}$	0.446	0.477	0.423
k_{off}^{P}	min^{-1}	181	196	169
$k_{forward}$	min^{-1}	22.8	25.0	21.9
k_{off}^{NP}	min^{-1}	0.281	0.331	0.19

Supplementary Table 4. Sample size and replicates for kinetic studies of WT and mutant Dis3L2 with different substrates

Exp. Number	Enzyme	Substrate	Enzyme titrations (RNA: 1 nM)	Replicates	Pulse-chase assay (RNA = 1 nM, Scavenger = 5000 nM)	Replicates
1	WT *hs*Dis3L2	HairpinA-GCU_{14}	Dis3L2: 5, 10, 12.5, 25, 50, 100, 250, 500 & 1000 nM	5	Dis3L2: 50 & 100 nM	4
2	WT *hs*Dis3	HairpinA-GCU_{14}	Dis3: 0.1, 0.5, 1, 5, 10, 25, 50, 100 & 250 nM	2	Dis3: 5 & 10 nM	3
3	ΔCSD Dis3L2	HairpinA-GCU_{14}	ΔCSD: 5, 10, 25, 50, 100, 250, 500 & 1000 nM	2	ΔCSD: 50 & 100 nM	3
4	Δ123H Dis3L2	HairpinA-GCU_{14}	Δ123H: 5, 10, 12.5, 25, 50, 100, 250, 500 & 1000 nM	3	Δ123H: 50 & 100 nM	4
5	WT *hs*Dis3L2	HairpinB-GCU_{14}	Dis3L2: 25, 50, 100, 250 & 500 nM	1	Dis3L2: 50 & 100 nM	2
6	WT *hs*Dis3L2	HairpinA-U_{16}	N/A	N/A	Dis3L2: 50 & 100 nM	2
7	WT *hs*Dis3L2	PolyU_{34}	Dis3L2: 10, 25, 50, 100 & 250 nM	2	Dis3L2: 50 & 100 nM	2
8	Δ123H Dis3L2	PolyU_{34}	Δ123H: 50 & 250 nM	1	N/A	N/A
9	WT *hs*Dis3L2	7SL-HairpinF-U_{16}	Dis3L2: 50, 100 & 200 nM	1	Dis3L2: 50 & 100 nM	2
10	WT *hs*Dis3L2	7SL-HairpinG-U_{16}	Dis3L2: 50, 100 & 200 nM	1	Dis3L2: 50 & 100 nM	2
11	WT *hs*Dis3L2	HairpinI-GCU_{14}	Dis3L2: 50, 100 & 200 nM	1	Dis3L2: 50 & 100 nM	2
12	Δ1H Dis3L2	HairpinA-GCU_{14}	Δ1H: 5, 10, 12.5, 25, 50, 100, 250, 500 & 1000 nM	2	Δ1H: 50 & 100 nM	3
13	Δ2H Dis3L2	HairpinA-GCU_{14}	Δ2H: 5, 10, 12.5, 25, 50, 100, 250, 500 & 1000 nM	2	Δ2H: 50 & 100 nM	3
14	Δ3H Dis3L2	HairpinA-GCU_{14}	Δ3H: 5, 10, 12.5, 25, 50, 100, 250, 500 & 1000 nM	2	Δ3H: 50 & 100 nM	3

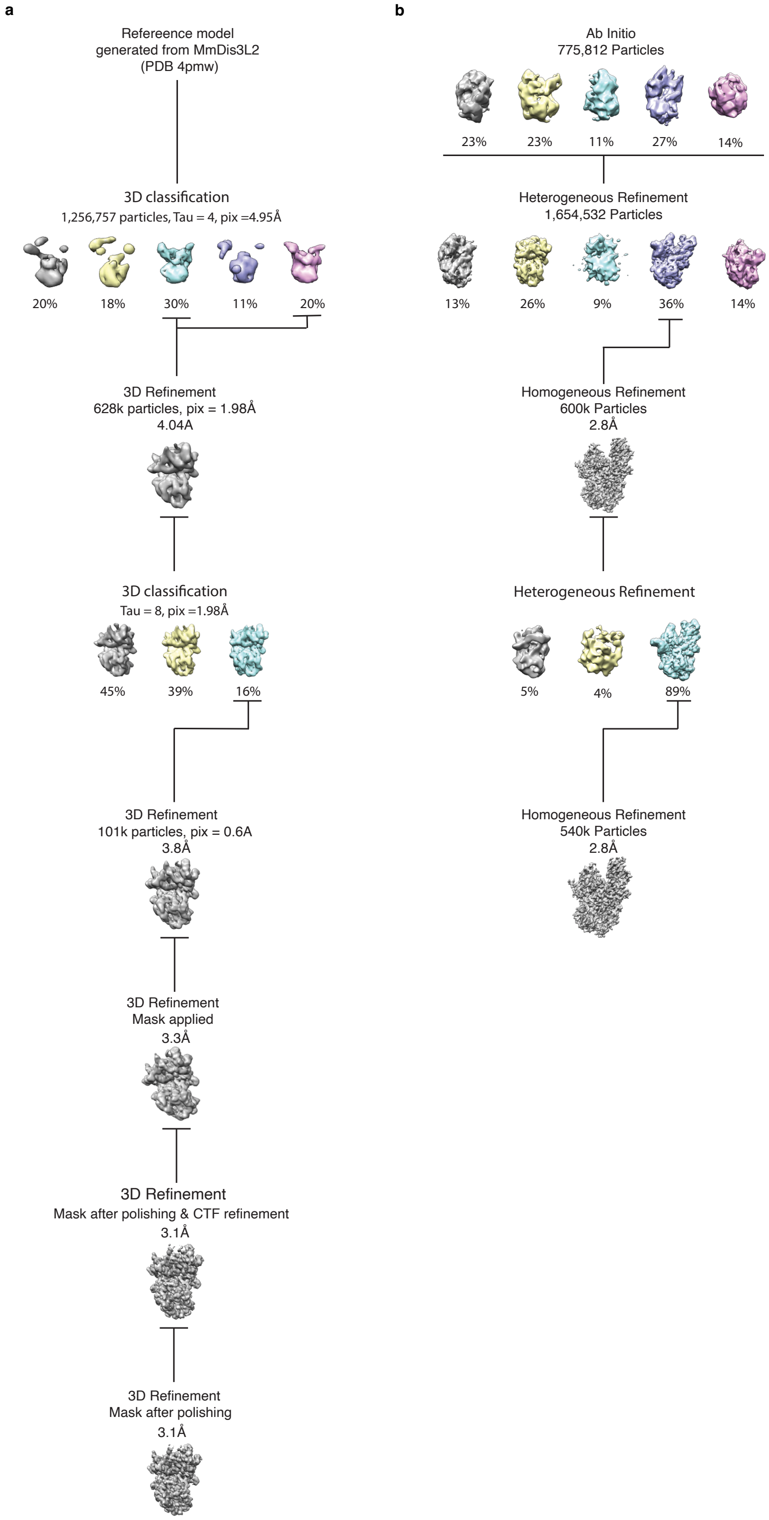

Supplementary Figure 1. Representative cryoEM data processing pathways. All datasets were pre-processed in WarpEM including motion correction, CTF estimation and particle picking. Further processing was done in Relion or CryoSPARC. **a**, Data processing of HsDis3L2-hairpinA-GCU$_{14}$ dataset in Relion. Following 2D classification to remove junk/bad particles the structure of MmDis3L2 was used to generate a starting model, and filtered to 20Å. A

series of refinement steps were then done to arrive at the final 3.1 Å resolution model used to build the HsDis3L2-hairpinA-GCU$_{14}$ structure. **b**, Data processing of the HsDis3L2-hairpinD-U$_7$ dataset was done in CryoSPARC. Following 2D classification, a subset of the best particles (as determined by high resolution estimation and high particle number in the 2D classes) were used for *ab initio* reconstruction of 5 classes. The resulting models were then used as starting references in a heterogeneous refinement using all the good particles picked by WarpEM. The highest resolution class was further refined in hetero- and homogeneous refinements to a final resolution of 2.8 Å.

Supplementary Figure 2. CryoEM Representative Micrograph of RNA-free HsDis3L2^{D391N}

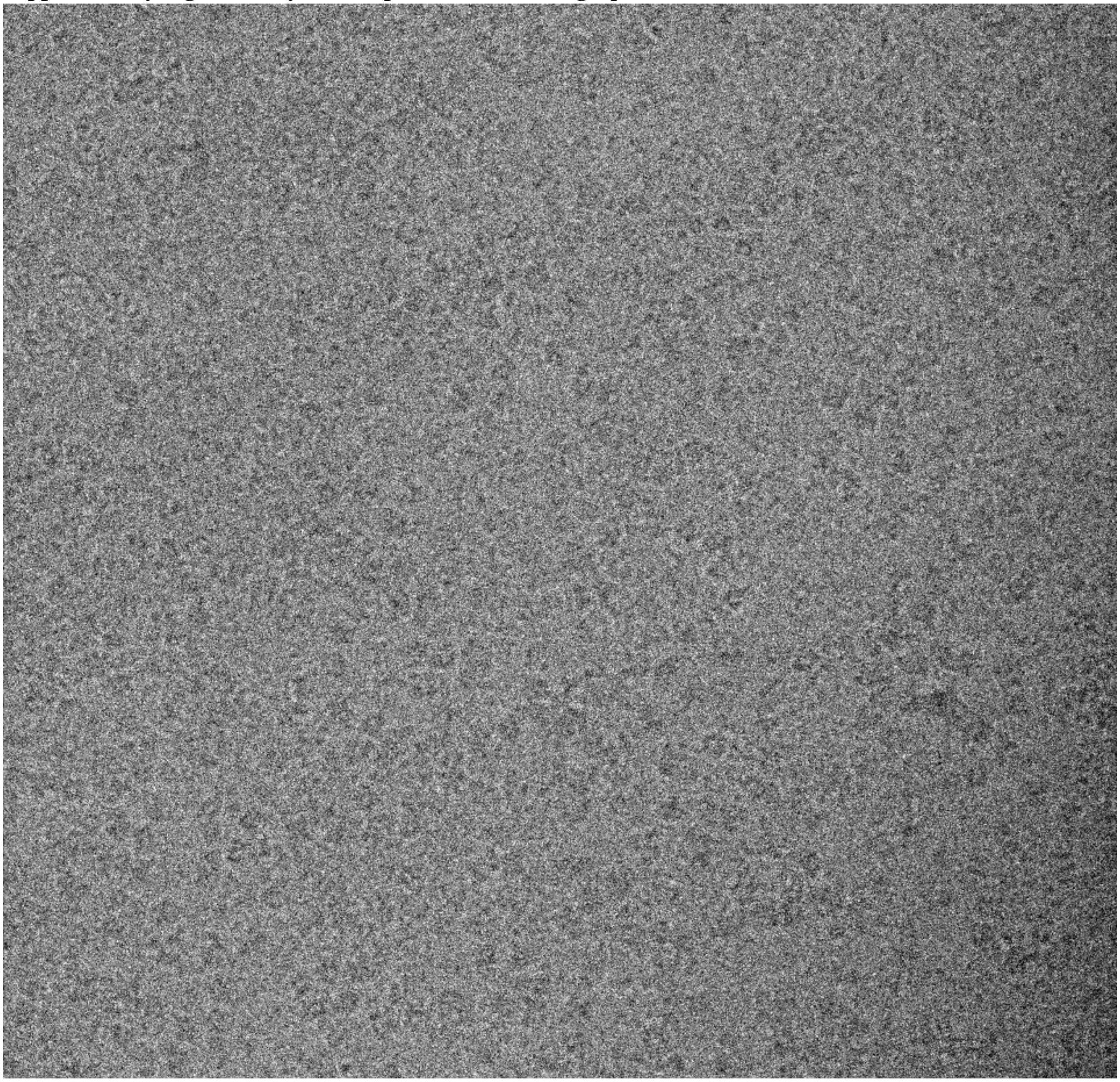

Supplementary Figure 3. CryoEM Representative Micrograph of RNA-free WT HsDis3L2

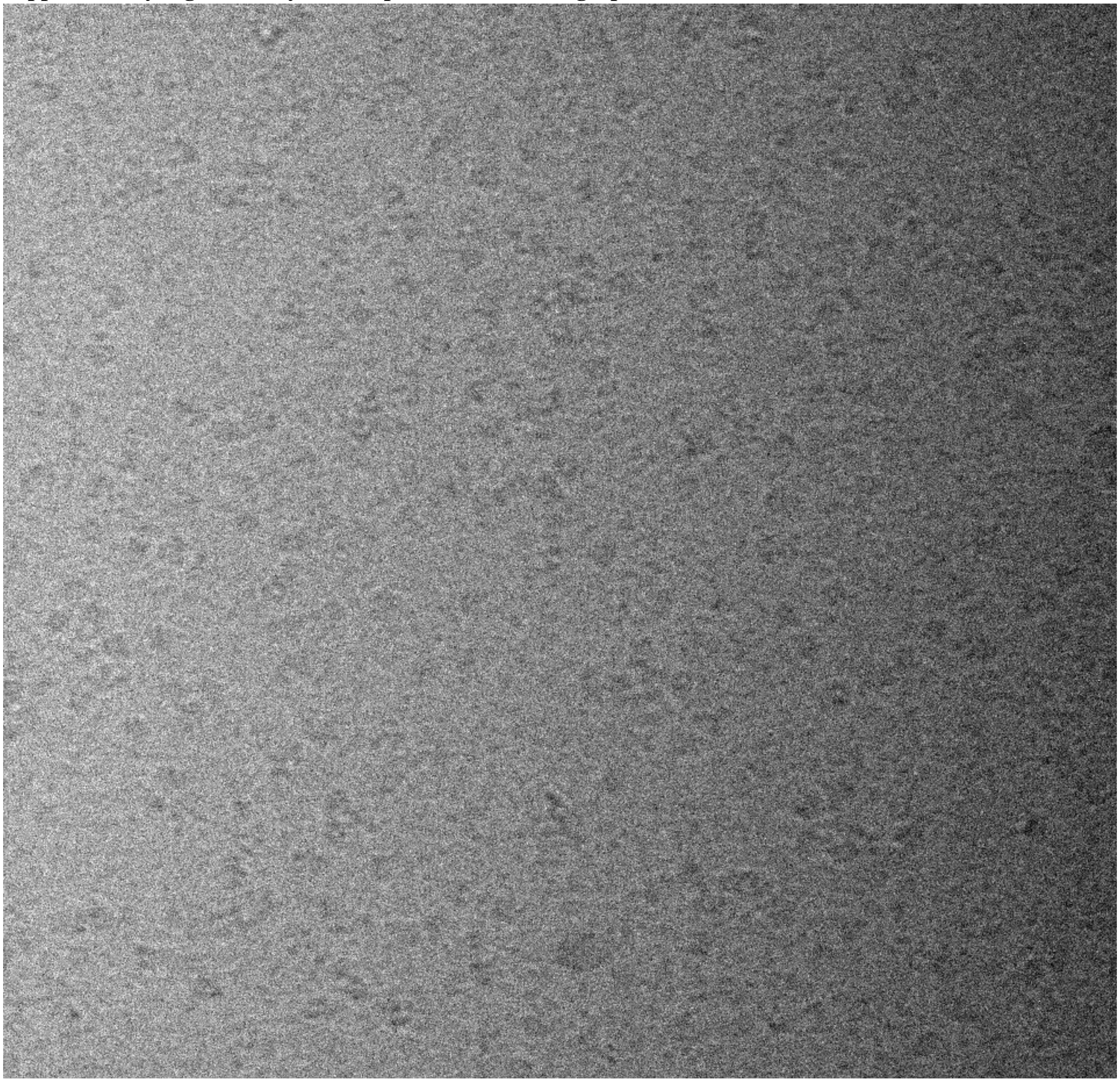

Supplementary Figure 4. CryoEM Representative Micrograph of WT HsDis3L2^{D391N} HairpinA-GCU$_{14}$

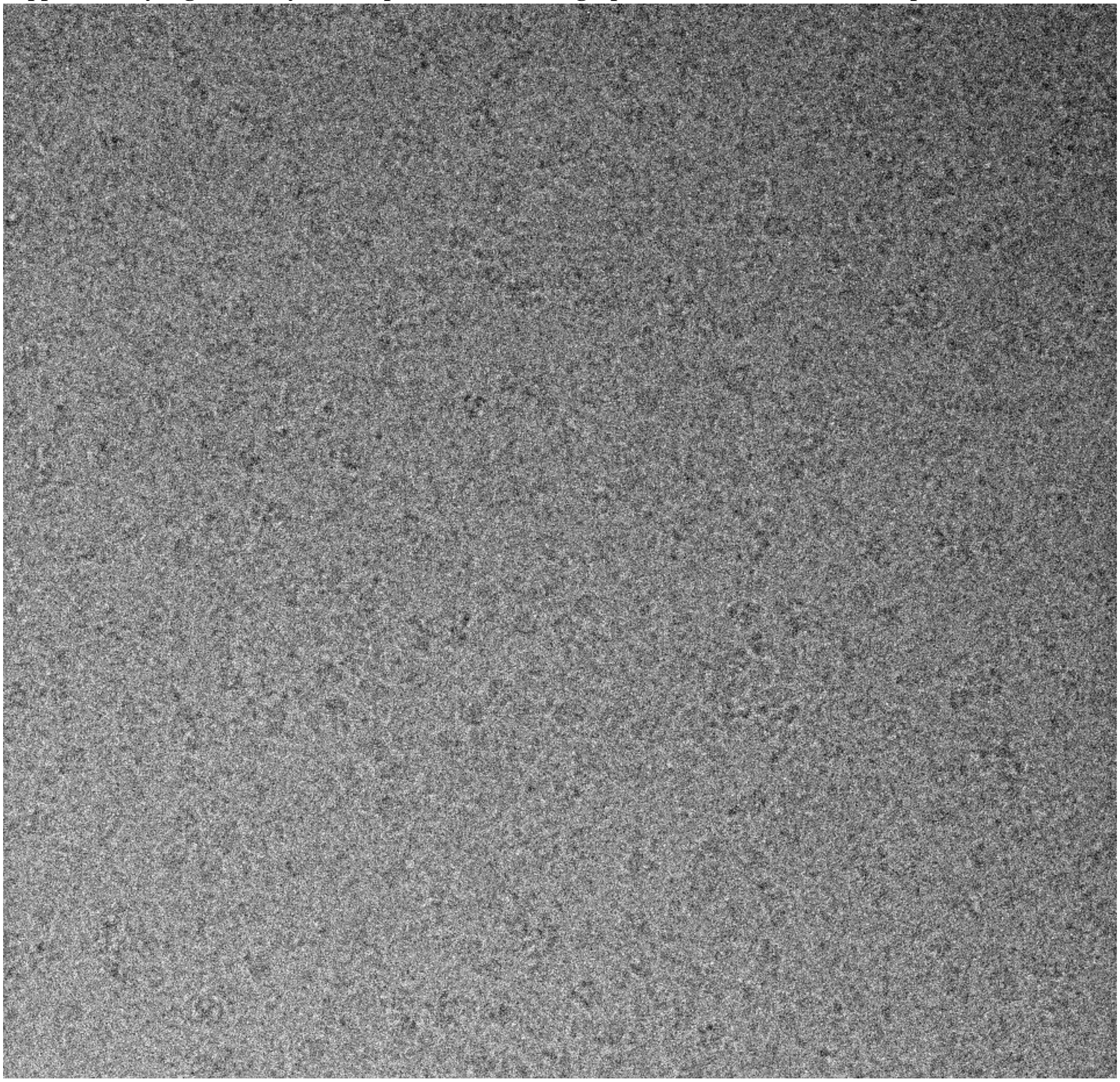

Supplementary Figure 5. CryoEM Representative Micrograph of WT HsDis3L2 HairpinC-U_{12}

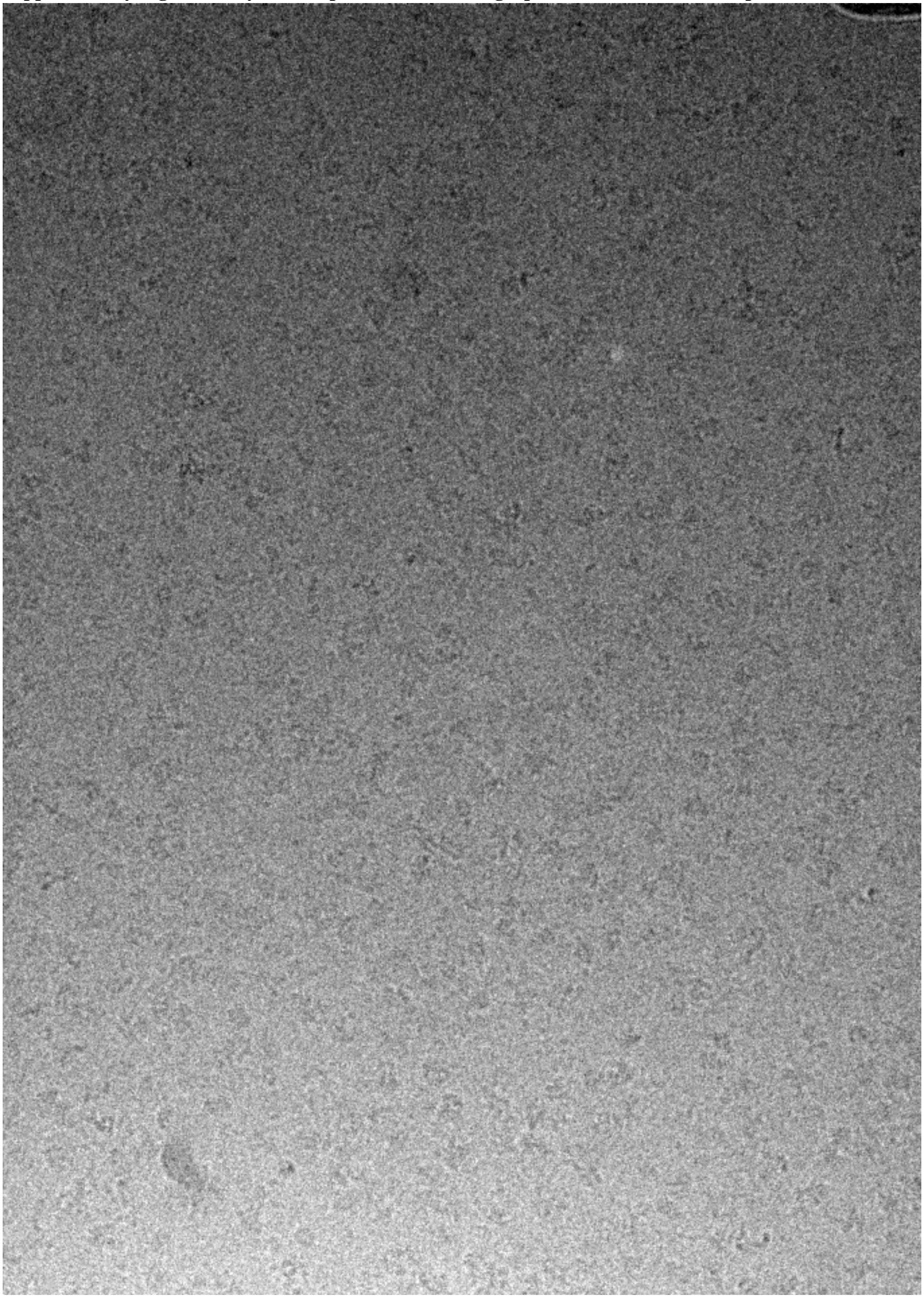

Supplementary Figure 6. CryoEM Representative Micrograph of WT HsDis3L2 HairpinD-U$_9$

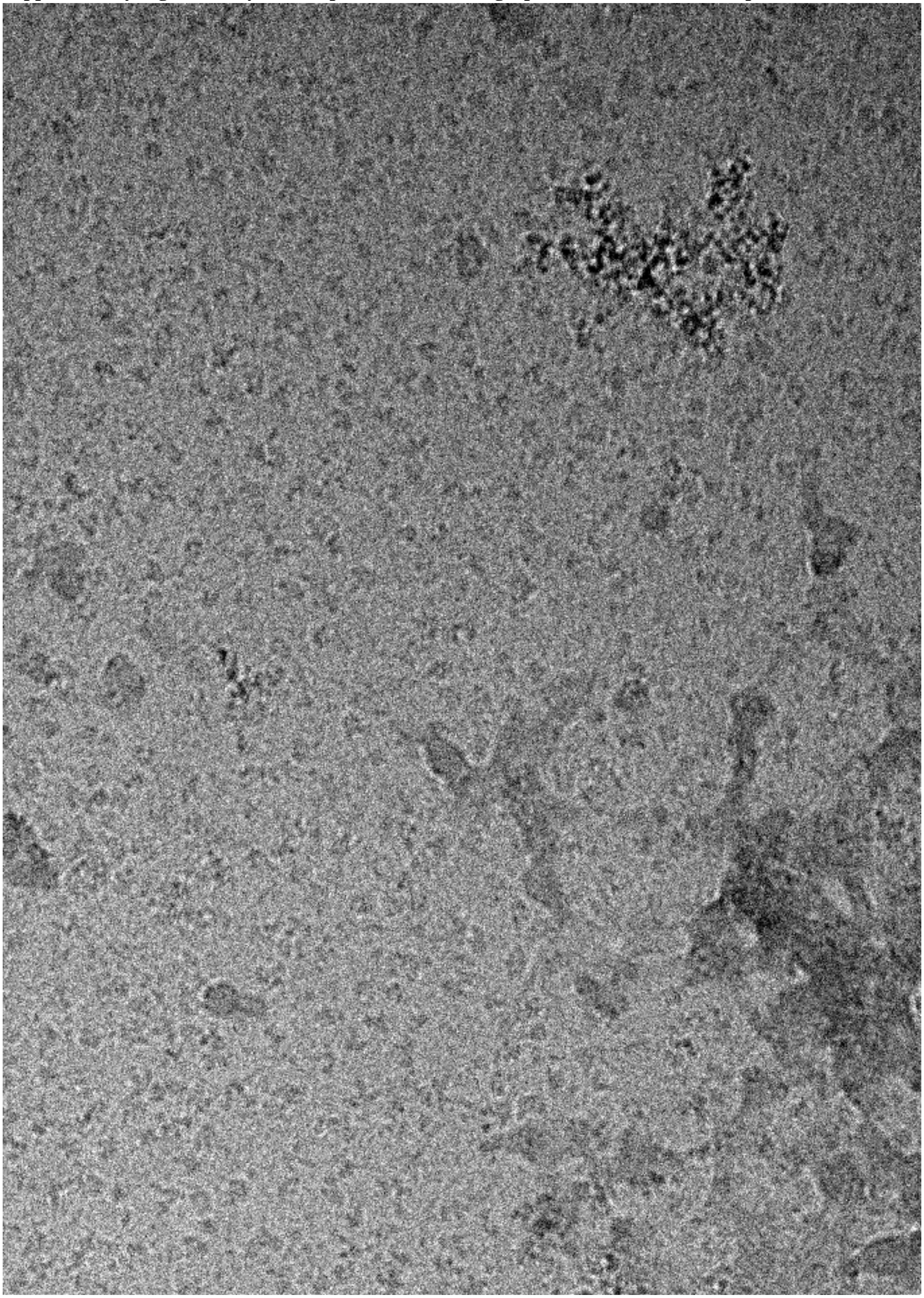

Supplementary Figure 7. CryoEM Representative Micrograph of WT HsDis3L2 HairpinD-U_9 #2

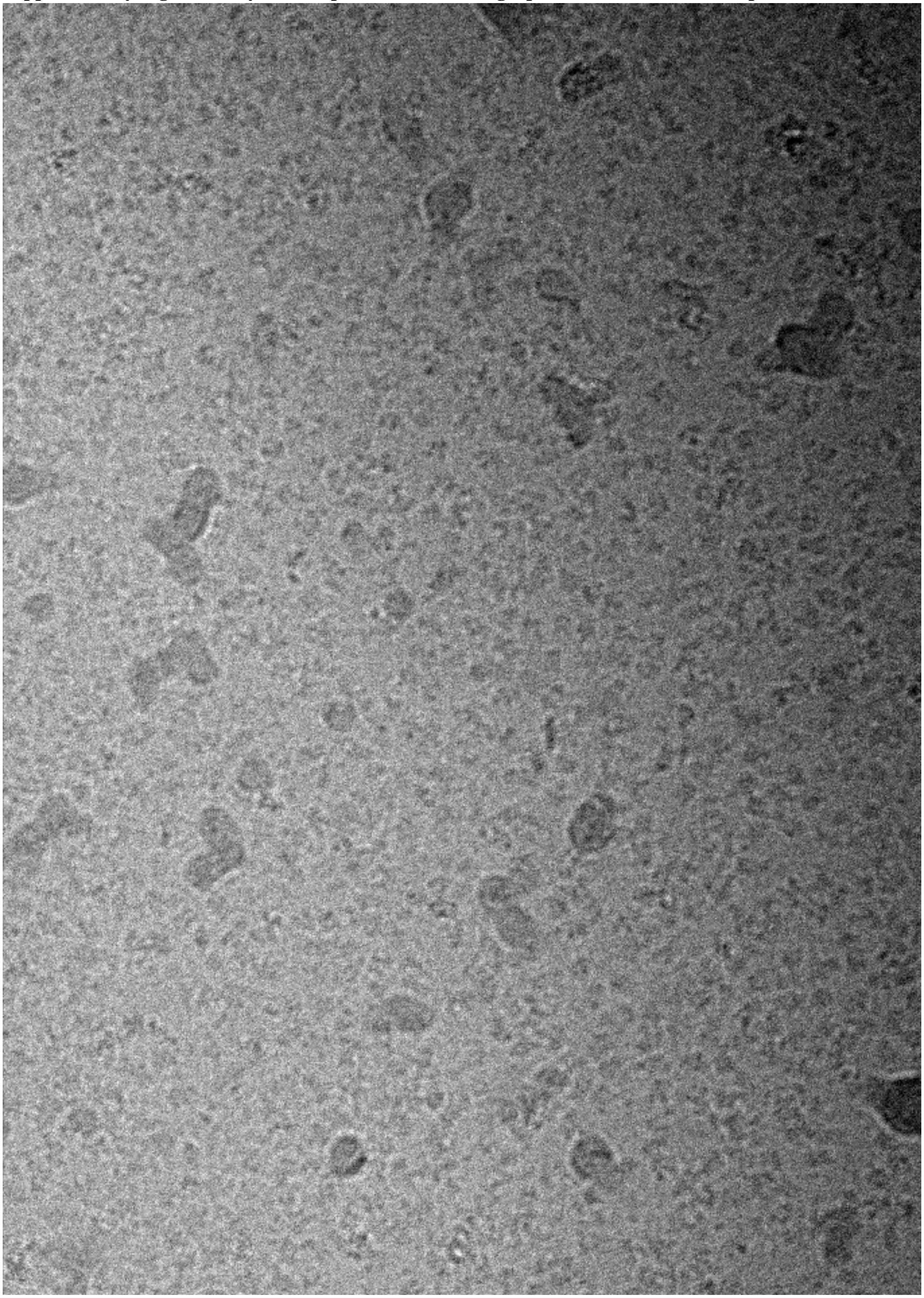

Supplementary Figure 8. CryoEM Representative Micrograph of WT HsDis3L2 HairpinD-U_8

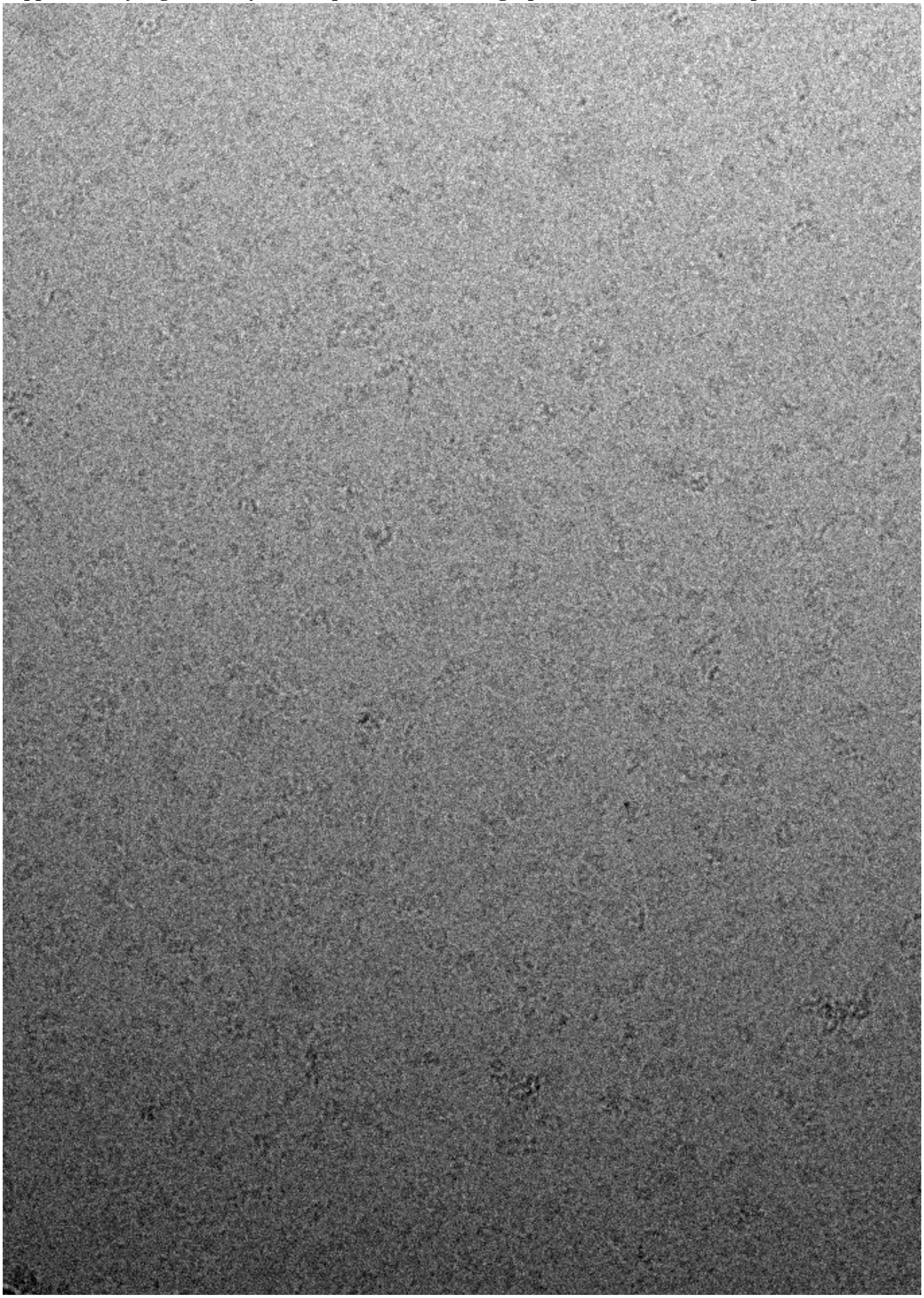

Supplementary Figure 9. CryoEM Representative Micrograph of WT HsDis3L2 HairpinD-U_8 #2

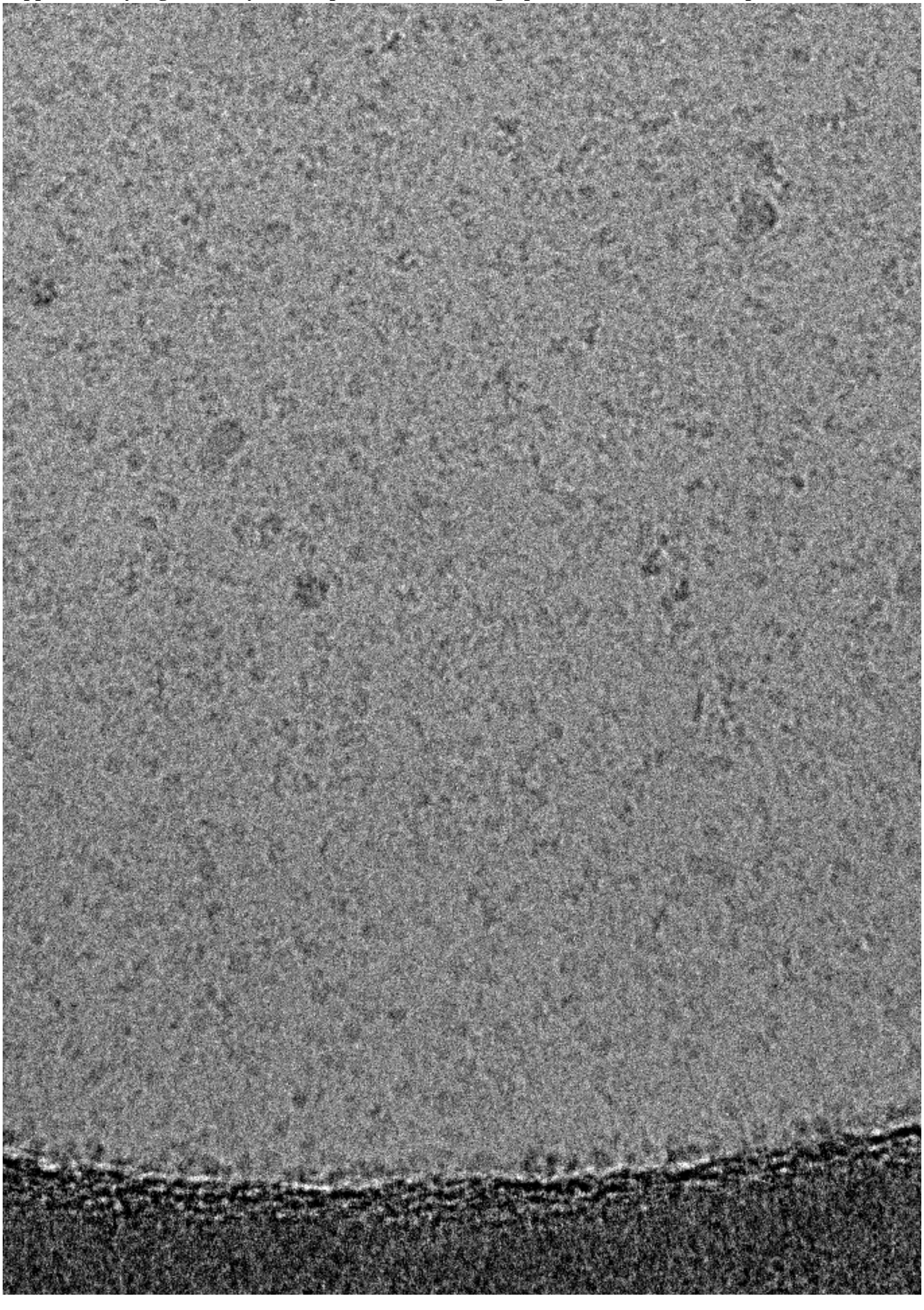

Supplementary Figure 10. CryoEM Representative Micrograph of WT HsDis3L2 HairpinD-U_9 +EDTA

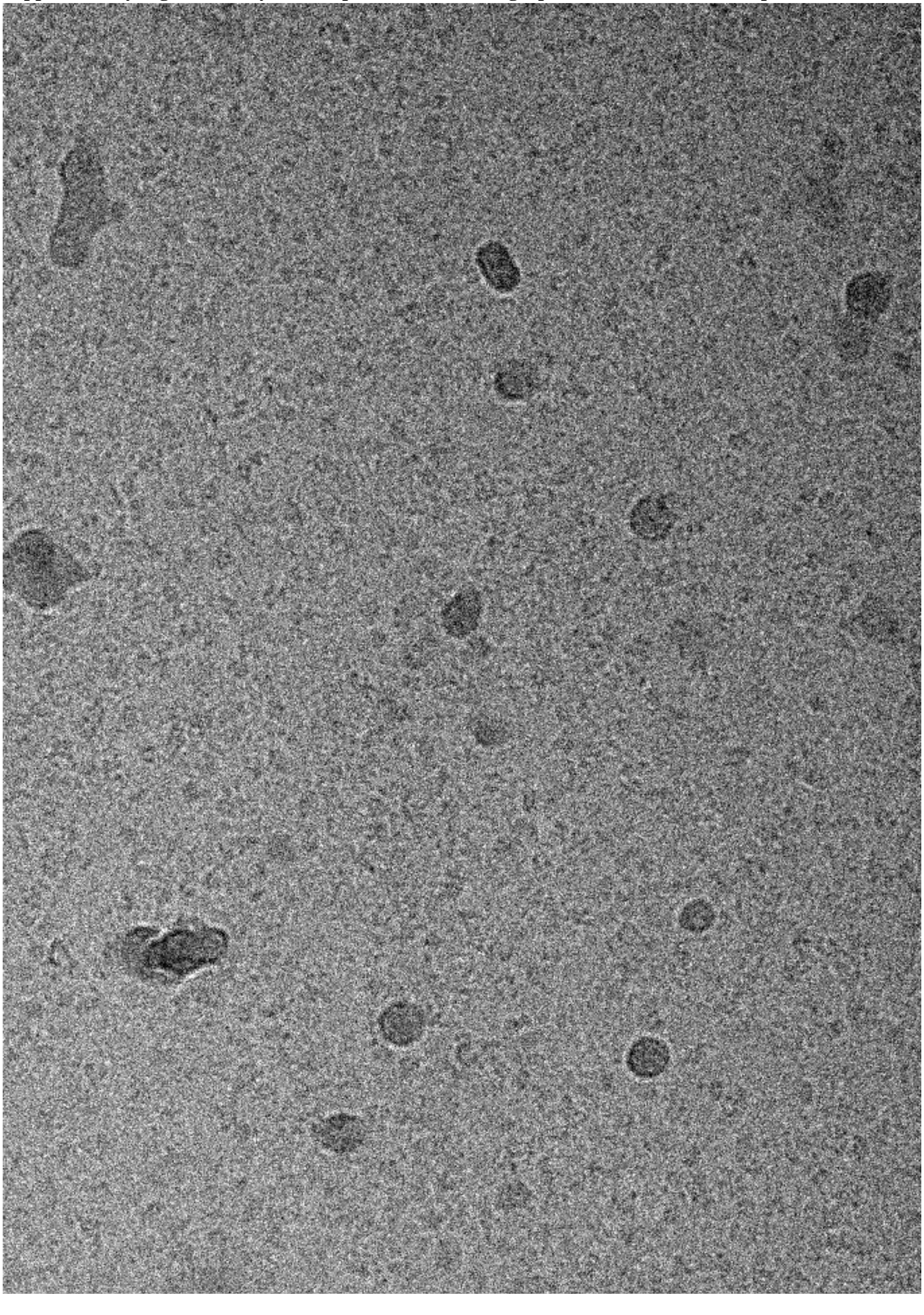

Supplementary Figure 11. CryoEM Representative Micrograph of WT HsDis3L2 HairpinD-U_7

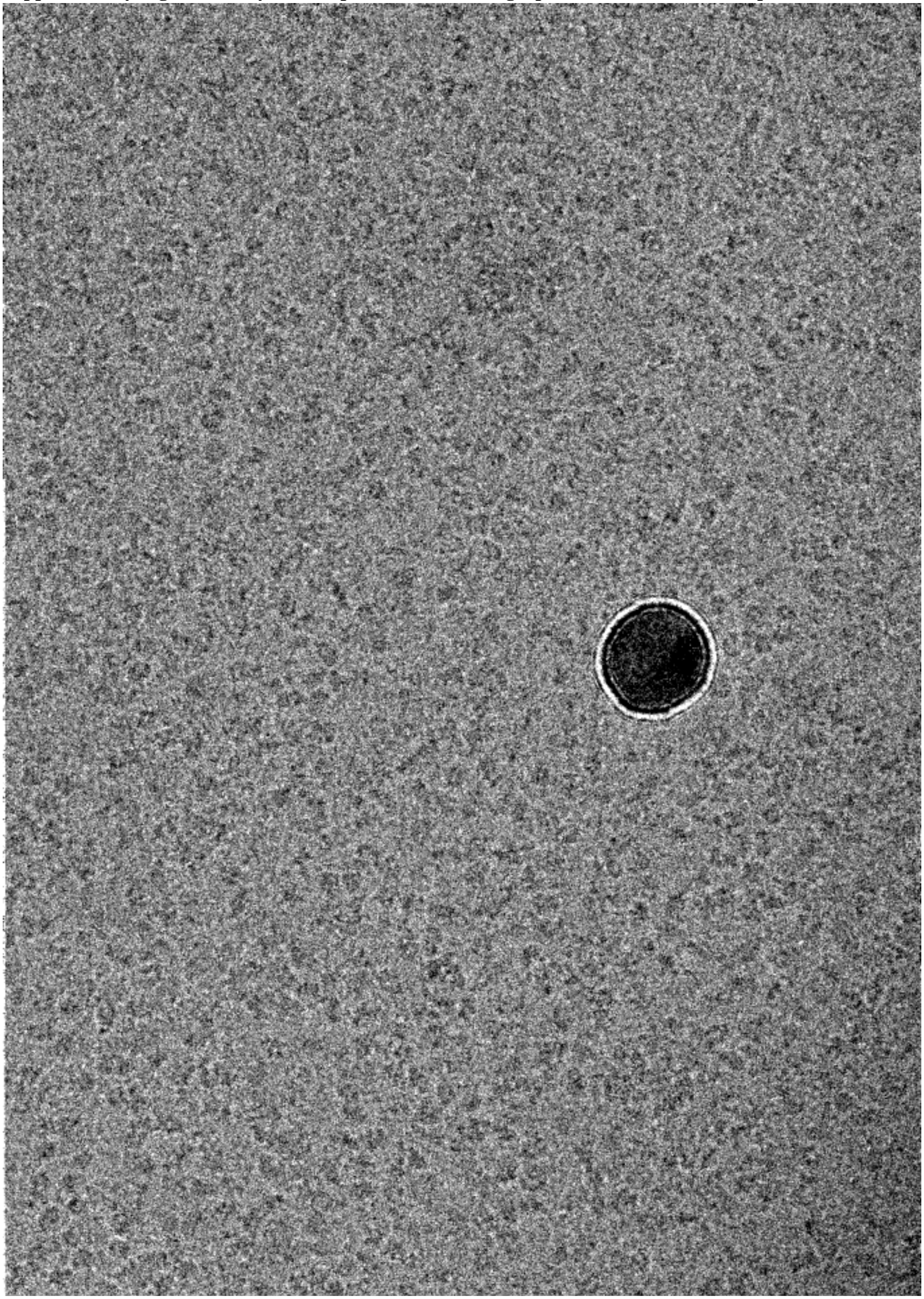

Supplementary Figure 12. CryoEM Representative Micrograph of WT HsDis3L2 HairpinD-U_7 #2

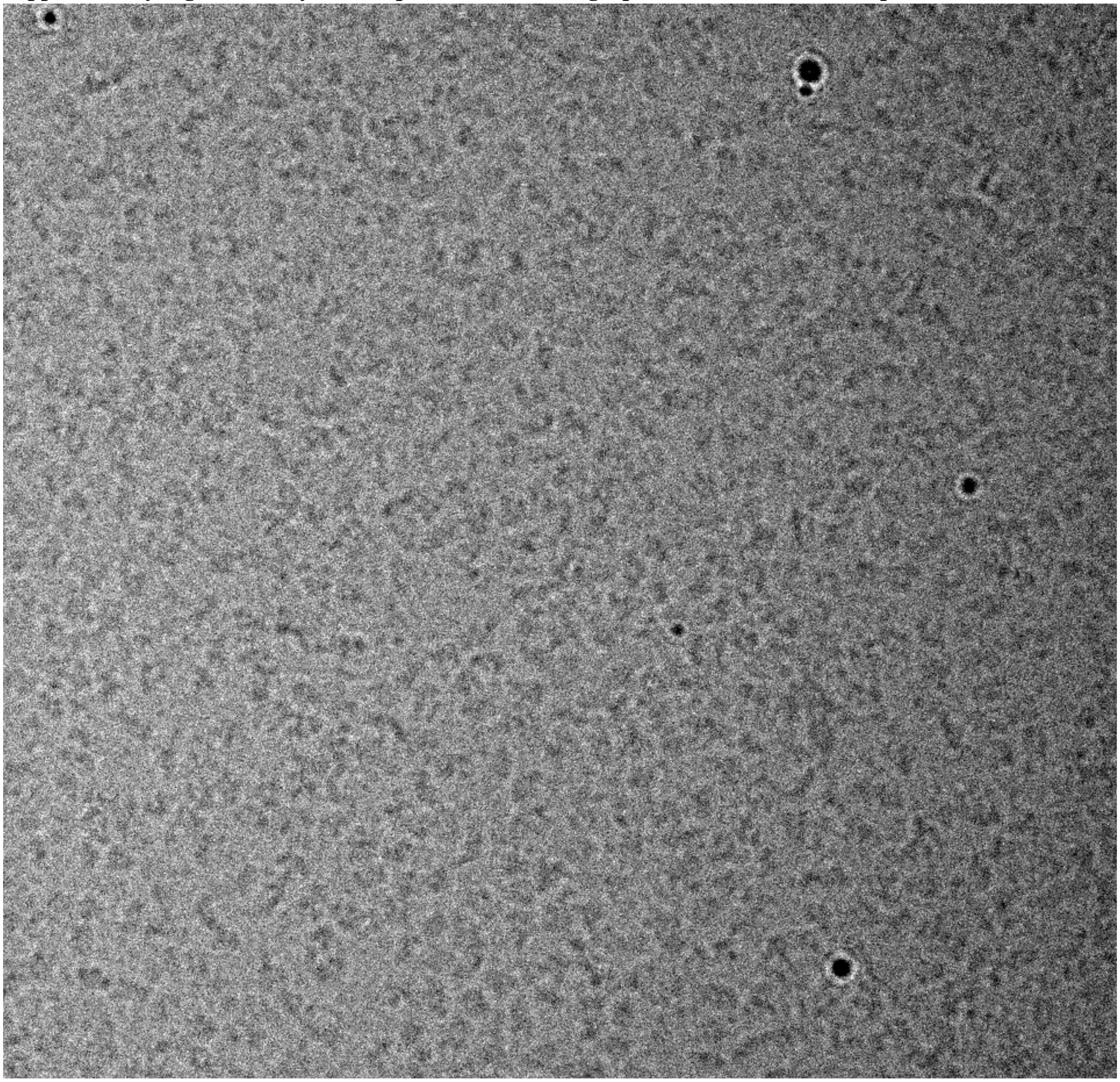

Supplementary Figure 13. CryoEM Representative Micrograph of WT HsDis3L2 HairpinD-U_5

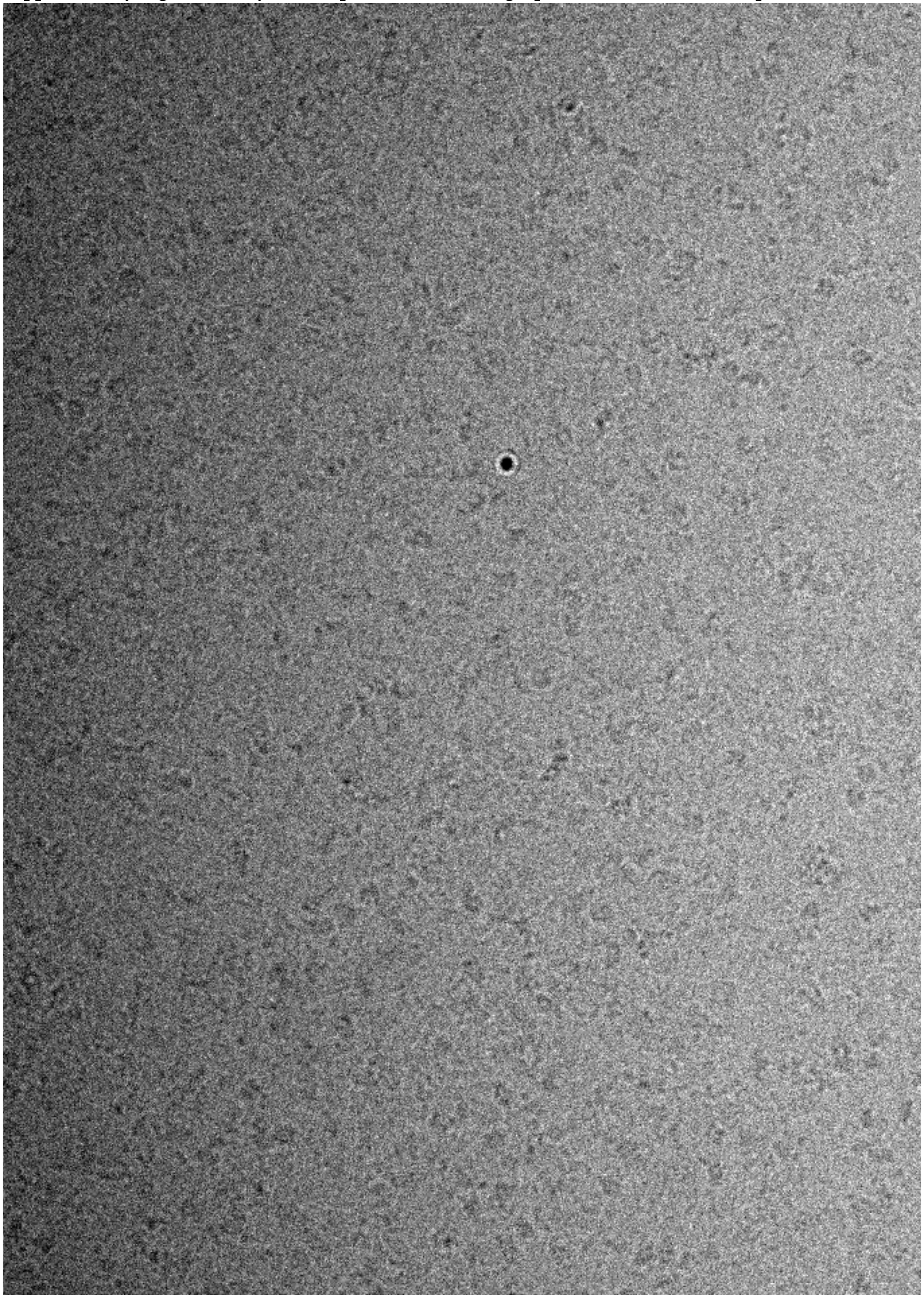

Supplementary Figure 14. CryoEM Representative Micrograph of WT HsDis3L2 HairpinD-U_5 +EDTA

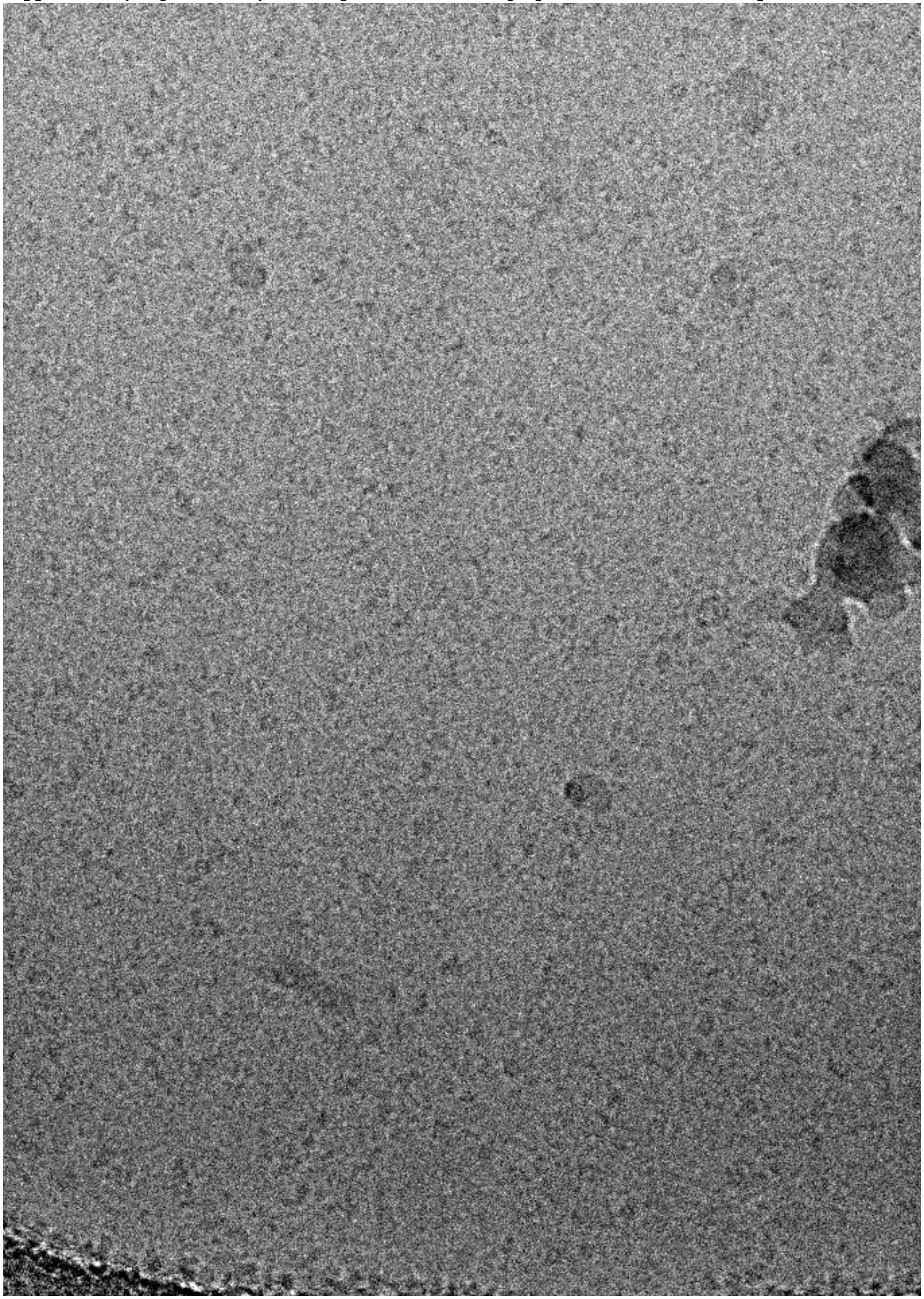

Supplement: Supplementary file 1 — Supplementary Tables 1–4 and Figs. 1–14. [file 41594_2023_923_MOESM1_ESM.pdf]
