# Supplementary material for: A shape-shifting nuclease unravels structured RNA
Source: Nat Struct Mol Biol. 2023 Feb 23;30(3):339–47. doi: 10.1038/s41594-023-00923-x (PMC10023572; doi:10.1038/s41594-023-00923-x)
Supplement: Supplementary file 2 — Reporting Summary [file 41594_2023_923_MOESM2_ESM.pdf]

nature portfolio

Corresponding author(s): Leemor Joshua-Tor

Last updated by author(s): Oct 16, 2022

Reporting Summary

Nature Portfolio wishes to improve the reproducibility of the work that we publish. This form provides structure for consistency and transparency in reporting. For further information on Nature Portfolio policies, see our Editorial Policies and the Editorial Policy Checklist.

Statistics

For all statistical analyses, confirm that the following items are present in the figure legend, table legend, main text, or Methods section.

n/a	Confirmed	
☐	☒	The exact sample size (*n*) for each experimental group/condition, given as a discrete number and unit of measurement
☐	☒	A statement on whether measurements were taken from distinct samples or whether the same sample was measured repeatedly
☒	☐	The statistical test(s) used AND whether they are one- or two-sided<br>*Only common tests should be described solely by name; describe more complex techniques in the Methods section.*
☒	☐	A description of all covariates tested
☐	☒	A description of any assumptions or corrections, such as tests of normality and adjustment for multiple comparisons
☐	☒	A full description of the statistical parameters including central tendency (e.g. means) or other basic estimates (e.g. regression coefficient) AND variation (e.g. standard deviation) or associated estimates of uncertainty (e.g. confidence intervals)
☒	☐	For null hypothesis testing, the test statistic (e.g. *F*, *t*, *r*) with confidence intervals, effect sizes, degrees of freedom and *P* value noted<br>*Give P values as exact values whenever suitable.*
☒	☐	For Bayesian analysis, information on the choice of priors and Markov chain Monte Carlo settings
☒	☐	For hierarchical and complex designs, identification of the appropriate level for tests and full reporting of outcomes
☒	☐	Estimates of effect sizes (e.g. Cohen's *d*, Pearson's *r*), indicating how they were calculated

Our web collection on statistics for biologists contains articles on many of the points above.

Software and code

Policy information about availability of computer code

Data collection	EPU ver 2.7 (ThermoFisher)
Data analysis	cryoEM: WarpEM ver 1.0.6 & 1.0.9 (Tegunov & Cramer); cryoSPARC v3.0.0 and v3.1.0 (Structura Biotechnology); Relion v 3.0 & 3.1 (Zivanov et al 2018) ; Coot 0.8.9.1 (Emsley & Cowtan 2004); Phenix 1.18-3855 (Afonine et al 2018, Terwilliger et al, 2020); Pymol 2.2.3 (Schrodinger, LLC); ChimeraX, ver 0.92 (RBVI);<br>kinetics: SAFA 1.0 (Das et al, 2005); Kinetic Explorer ver 8.0 (Kintek Global); GFIT 1.0 (Levin et al, 2009); Mathematica ver 6.0 (Wolfram); GraphPad Prism ver 9.1.2 (GraphPad Software).<br>other: FoRNA v1.0; Benchling (no version number, used 2019-2022); RNAfold (no version number).

For manuscripts utilizing custom algorithms or software that are central to the research but not yet described in published literature, software must be made available to editors and reviewers. We strongly encourage code deposition in a community repository (e.g. GitHub). See the Nature Portfolio guidelines for submitting code & software for further information.

Data

Policy information about availability of data

All manuscripts must include a data availability statement. This statement should provide the following information, where applicable:

- Accession codes, unique identifiers, or web links for publicly available datasets
- A description of any restrictions on data availability
- For clinical datasets or third party data, please ensure that the statement adheres to our policy

Structure coordinates and cryoEM data have been deposited in the PDB and EMDB respectively. The structures can be found under the following PDB accession numbers: RNA-free HsDis3L2: PDB 8E27, EMDB-27827; HsDis3L2 in complex with hairpinA-GCU14: PDB 8E28, EMDB-27828; HsDis3L2 in complex with hairpinC-U12: PDB 8E29, EMDB-27829; HsDis3L2 in complex with hairpinD-U17: PDB 8E2A, EMDB-27830. The cryoEM map of the low resolution HsDis3L2 complex with hairpinE-U7 was deposited in the EMDB under accession code EMDB-27831. The structure of mouse Dis3L2 (PDB 4PMW) was used as a reference and for comparisons. Source data for the kinetic analysis are provided with this paper.

Human research participants

Policy information about studies involving human research participants and Sex and Gender in Research.

Reporting on sex and gender	n/a
Population characteristics	n/a
Recruitment	n/a
Ethics oversight	n/a

Note that full information on the approval of the study protocol must also be provided in the manuscript.

Field-specific reporting

Please select the one below that is the best fit for your research. If you are not sure, read the appropriate sections before making your selection.

☒ Life sciences ☐ Behavioural & social sciences ☐ Ecological, evolutionary & environmental sciences

For a reference copy of the document with all sections, see nature.com/documents/nr-reporting-summary-flat.pdf

Life sciences study design

All studies must disclose on these points even when the disclosure is negative.

Sample size	For pulse-chase experiments, at least three replicate reactions were run at two different enzyme concentrations and the data were fit separately to analytical functions to calculate mean +/- standard deviation. For enzyme titration experiments, we tested at least five different enzyme concentrations with each reaction/concentration performed multiple times as indicated in the attached table.<br>No statistical methods were used to determine sample size. Instead, an appropriate sample size for enzyme titrations and pulse-chase experiments was picked to ensure a large enough dataset to obtain a global fit that yielded kinetic parameters that were well constrained by the data. Goodness of fit and quality of kinetic parameters were judged by examining the Std Error of Mean values and asymmetric error of the parameters via FitSpace analysis. All these values were well below the statistical thresholds that are customary in enzyme kinetic analysis.
Data exclusions	see Supplementary Table 4, which includes all requested information. Exclusions were made for kinetic data only due to large uncertainty
Replication	Kinetic experiments had a high degree of reproducibility as judged by: i) statistical parameters of mean +/- standard deviation for the analytical fits, ii) best fit parameters +/- standard errors of the mean for the global fits, and iii) asymmetric error analysis of kinetic parameters using the FitSpace feature of Kintek Explorer software.<br>All attempts of replication were successful.
Randomization	For the kinetic and structural analyses, there is no opportunity for randomization.
Blinding	For particle picking in the cryoEM experiments to determine structures, these were done using criteria in a non-biased way, with no subjective input.<br>Other than that there is no room for blinding in any of the experiments described, no clinical data are analyzed.

Reporting for specific materials, systems and methods

We require information from authors about some types of materials, experimental systems and methods used in many studies. Here, indicate whether each material, system or method listed is relevant to your study. If you are not sure if a list item applies to your research, read the appropriate section before selecting a response.

Materials & experimental systems

n/a	Involved in the study
☒	☐ Antibodies
☐	☒ Eukaryotic cell lines
☒	☐ Palaeontology and archaeology
☒	☐ Animals and other organisms
☒	☐ Clinical data
☒	☐ Dual use research of concern

Methods

n/a	Involved in the study
☒	☐ ChIP-seq
☒	☐ Flow cytometry
☒	☐ MRI-based neuroimaging

Eukaryotic cell lines

Policy information about cell lines and Sex and Gender in Research

Cell line source(s)	Spodoptera frugiperda: pupal ovarian cells (Sf9), Gibco / ThermoFisher Scientific<br>Catalogue Number: 11496015; RRID: CVCL_0549
Authentication	Cell line was not authenticated, it was used solely for the purpose of producing protein. The protein was validated using Mass Spectrometry.
Mycoplasma contamination	Cell line was not tested for contamination since correct protein was produced and validated.
Commonly misidentified lines<br>(See ICLAC register)	Not a commonly misidentified line.
