# Supplementary figures and images for: A shape-shifting nuclease unravels structured RNA
Source: Nat Struct Mol Biol. 2023 Feb 23;30(3):339–47. doi: 10.1038/s41594-023-00923-x (PMC10023572; doi:10.1038/s41594-023-00923-x)

ED Fig 4e, lanes 10-15

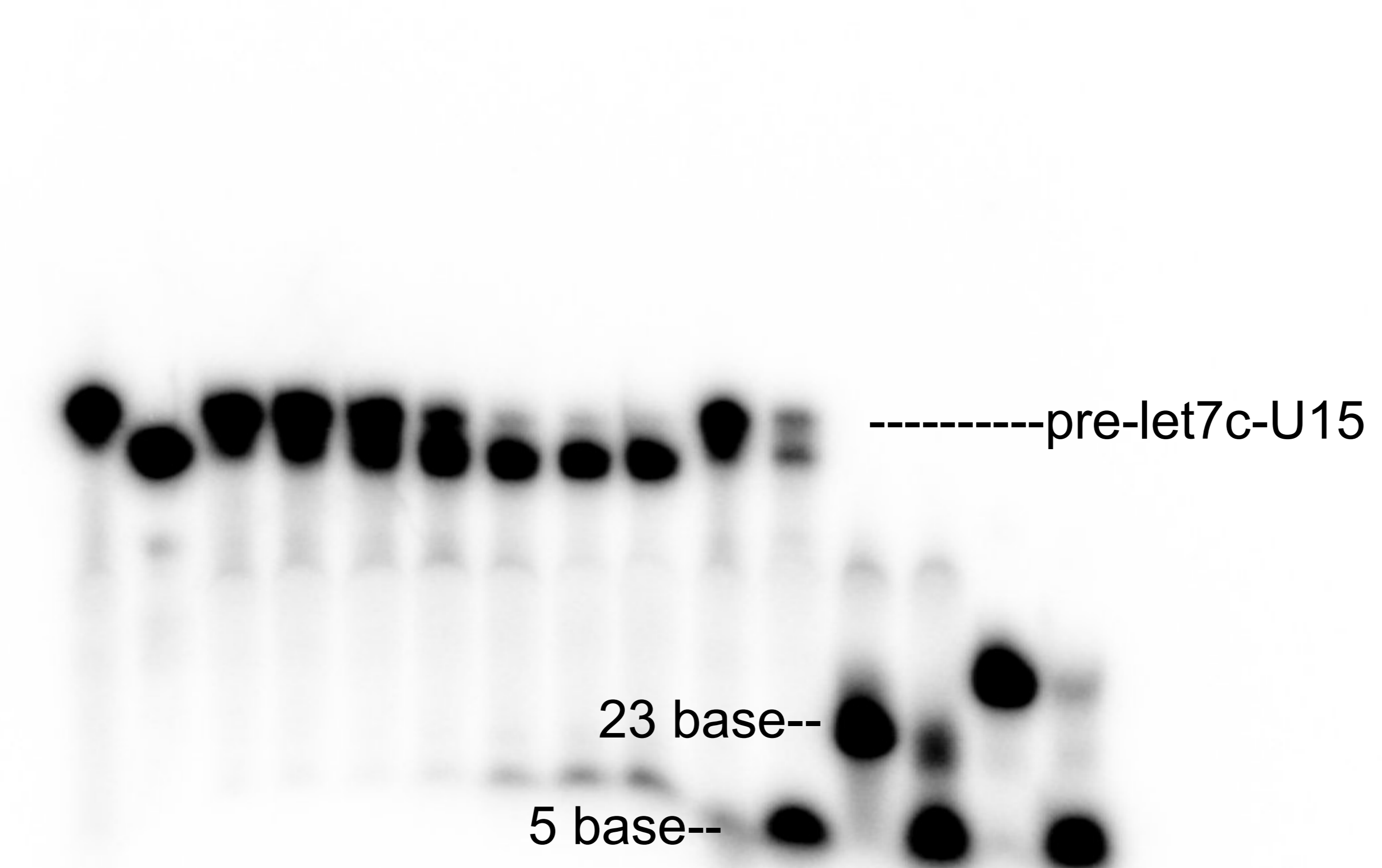

Supplement: Source Data Extended Data Fig. 4 — Uncropped gel for Extended Data Fig. 4e. [file 41594_2023_923_MOESM8_ESM.pdf]
