# Supplementary material for: A shape-shifting nuclease unravels structured RNA
Source: Nat Struct Mol Biol. 2023 Feb 23;30(3):339–47. doi: 10.1038/s41594-023-00923-x (PMC10023572; doi:10.1038/s41594-023-00923-x)

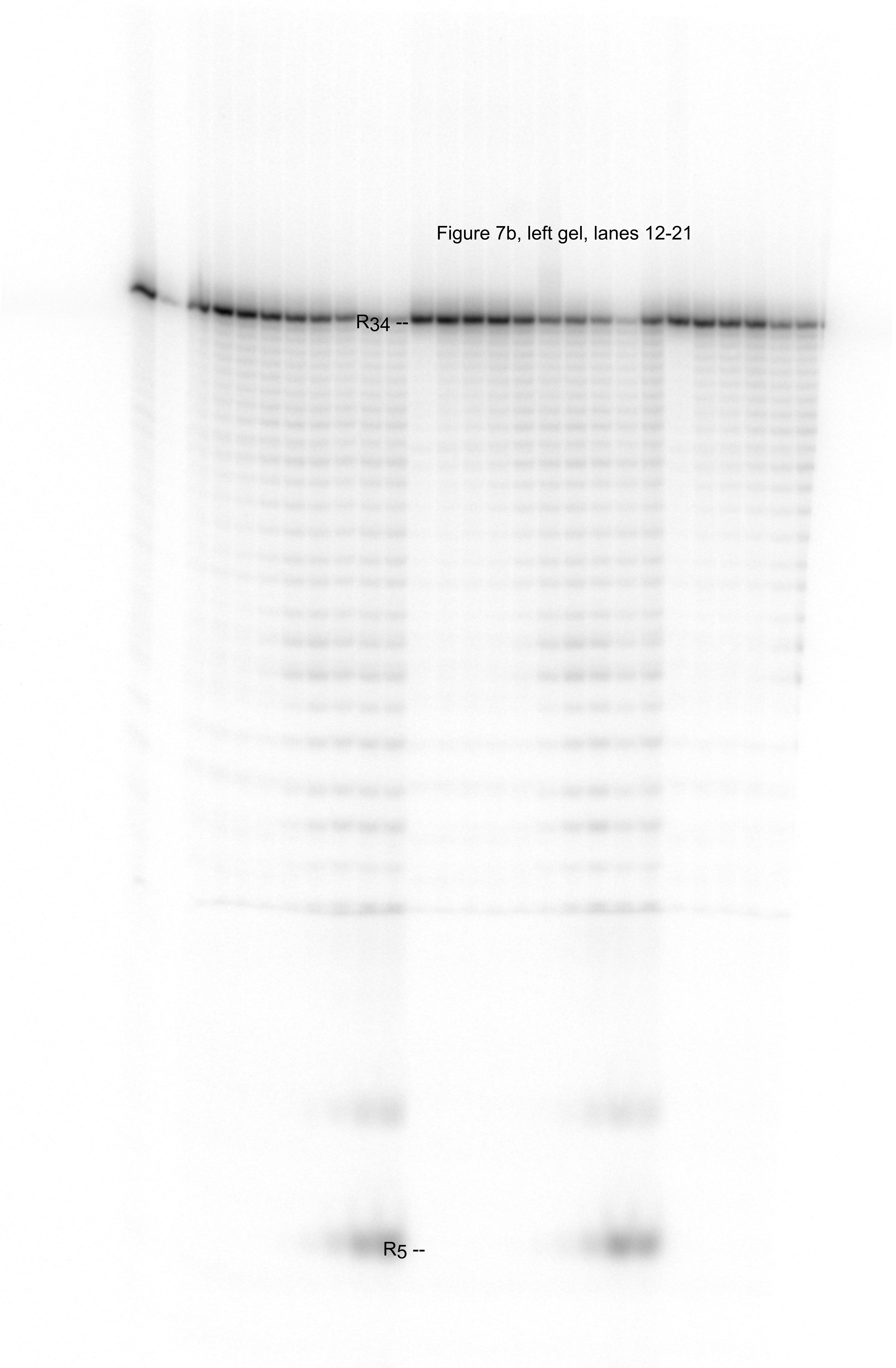
Figure 7b, left gel, lanes 12-21
R34 --
R5 --

Figure 7b, right gel, lanes 11-20

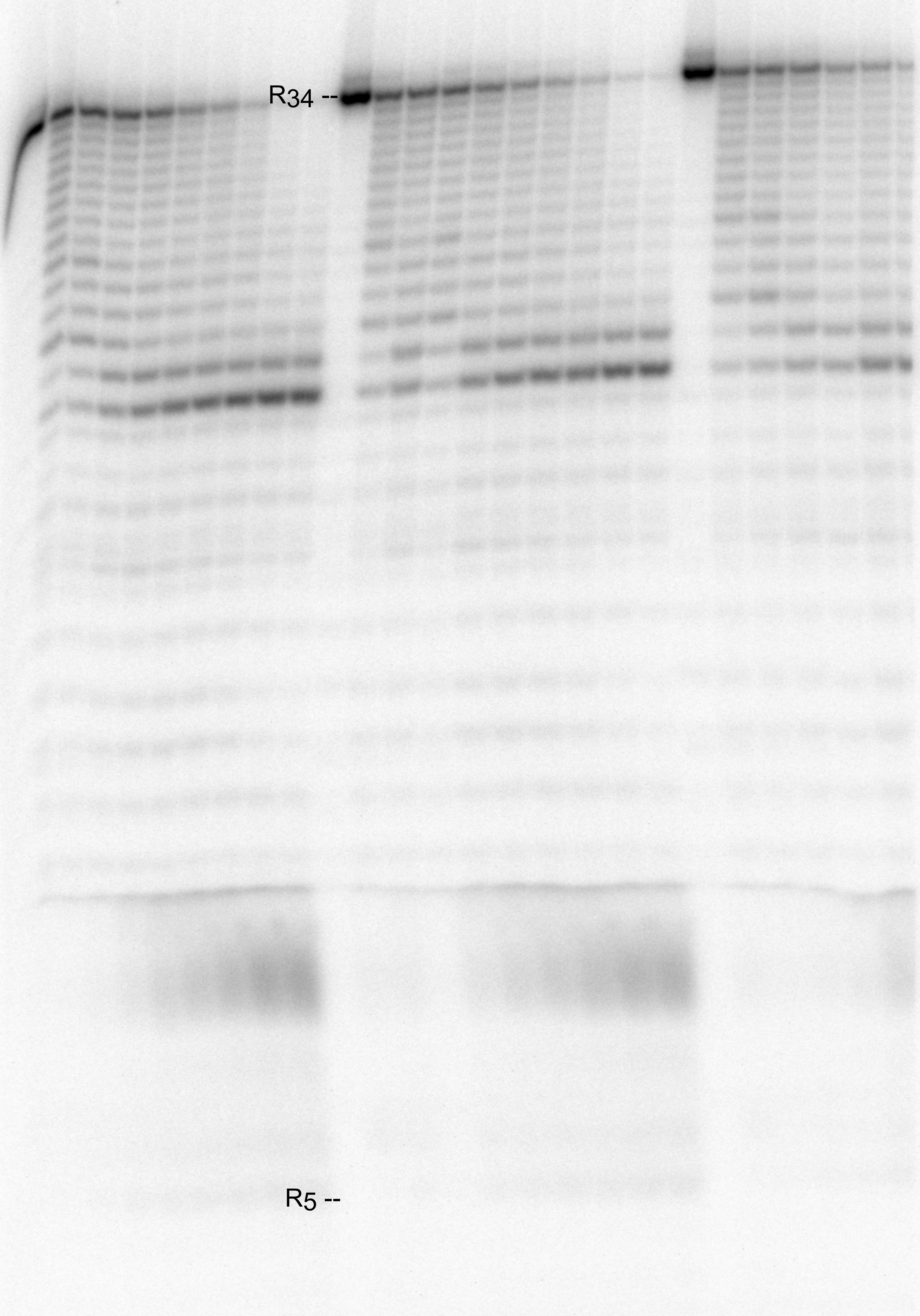

Figure 7 g, left gel, lanes 1-5

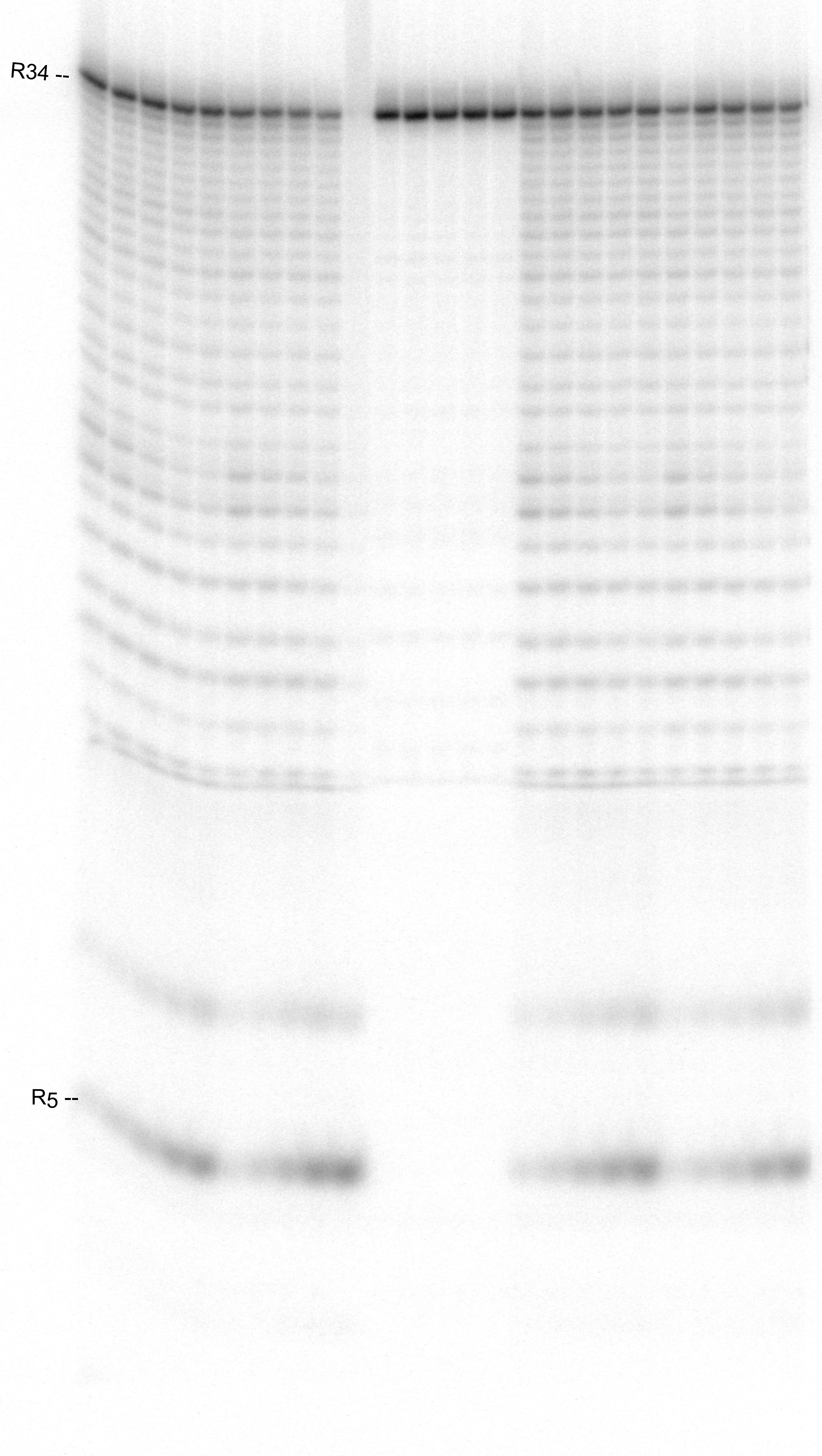

Figure 7g, middle gel, lanes 1-5

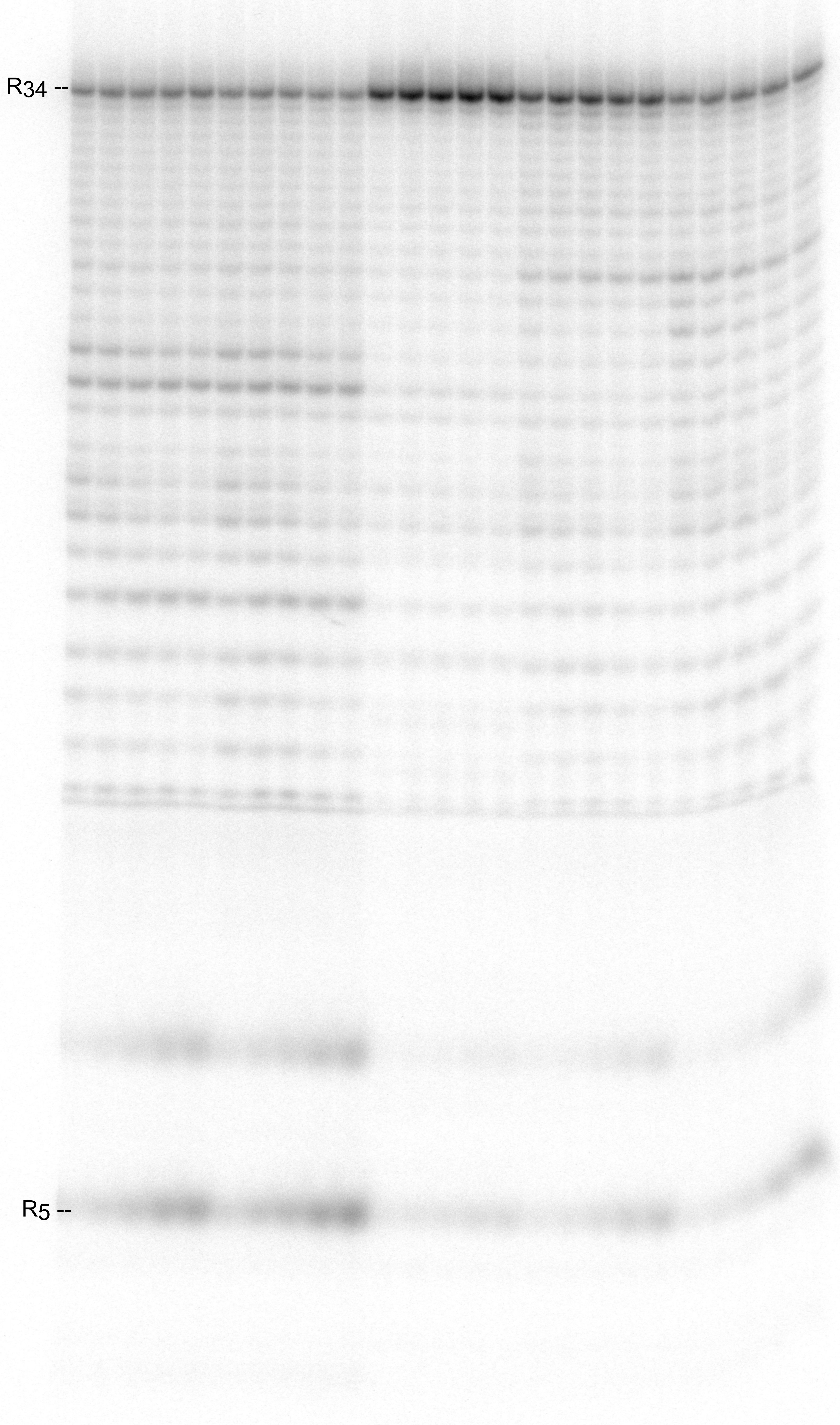

Figure 7g, right gel, lanes 1-5

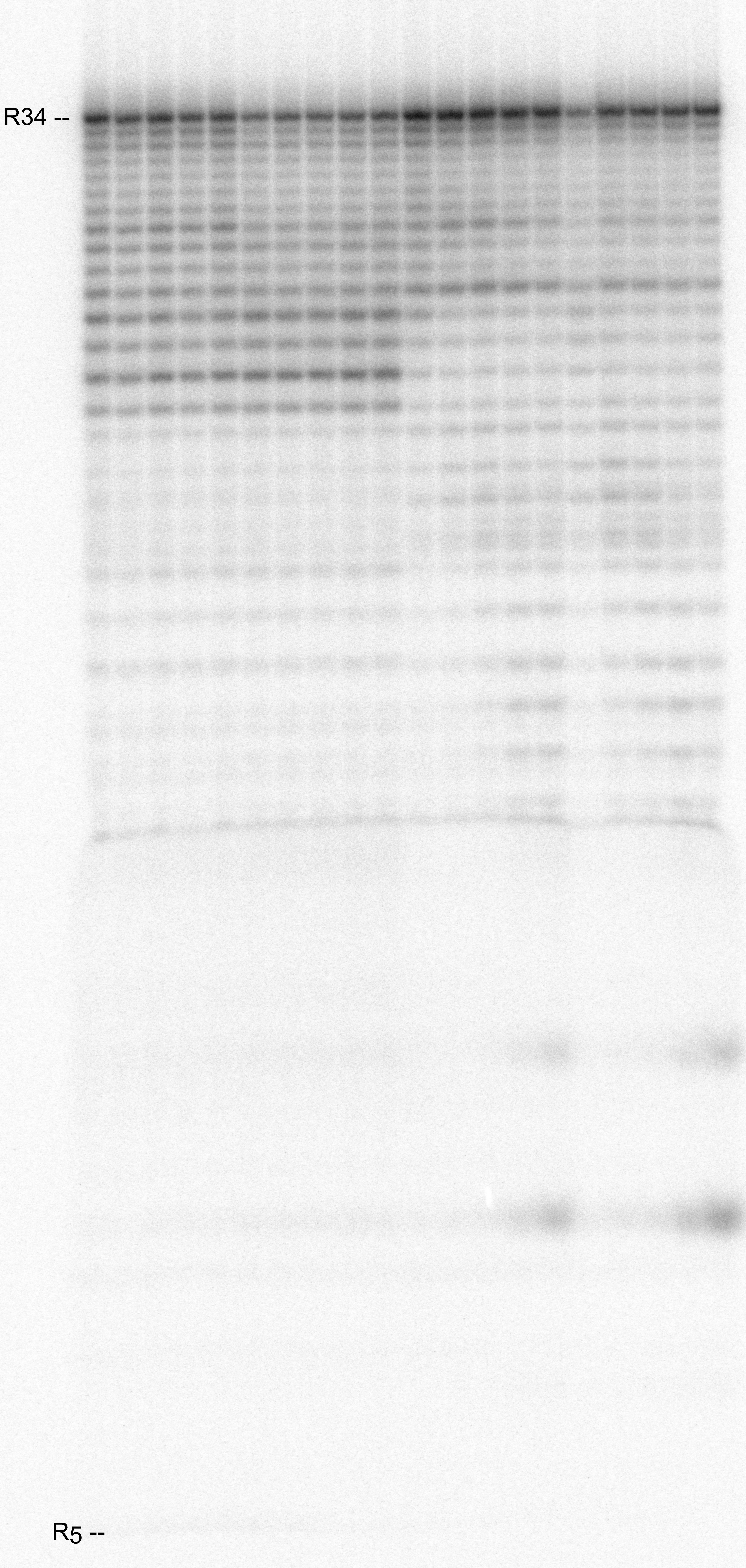

Supplement: Source Data Fig. 7 — Source data for calculated kinetic parameters and gels. [file 41594_2023_923_MOESM7_ESM.pdf]
