# Supplementary material for: A shape-shifting nuclease unravels structured RNA
Source: Nat Struct Mol Biol. 2023 Feb 23;30(3):339–47. doi: 10.1038/s41594-023-00923-x (PMC10023572; doi:10.1038/s41594-023-00923-x)
Supplement: Source Data Extended Data Fig. 7 — Source data (gels) for calculated kinetic parameters. [file 41594_2023_923_MOESM11_ESM.pdf]

ED Fig 7b

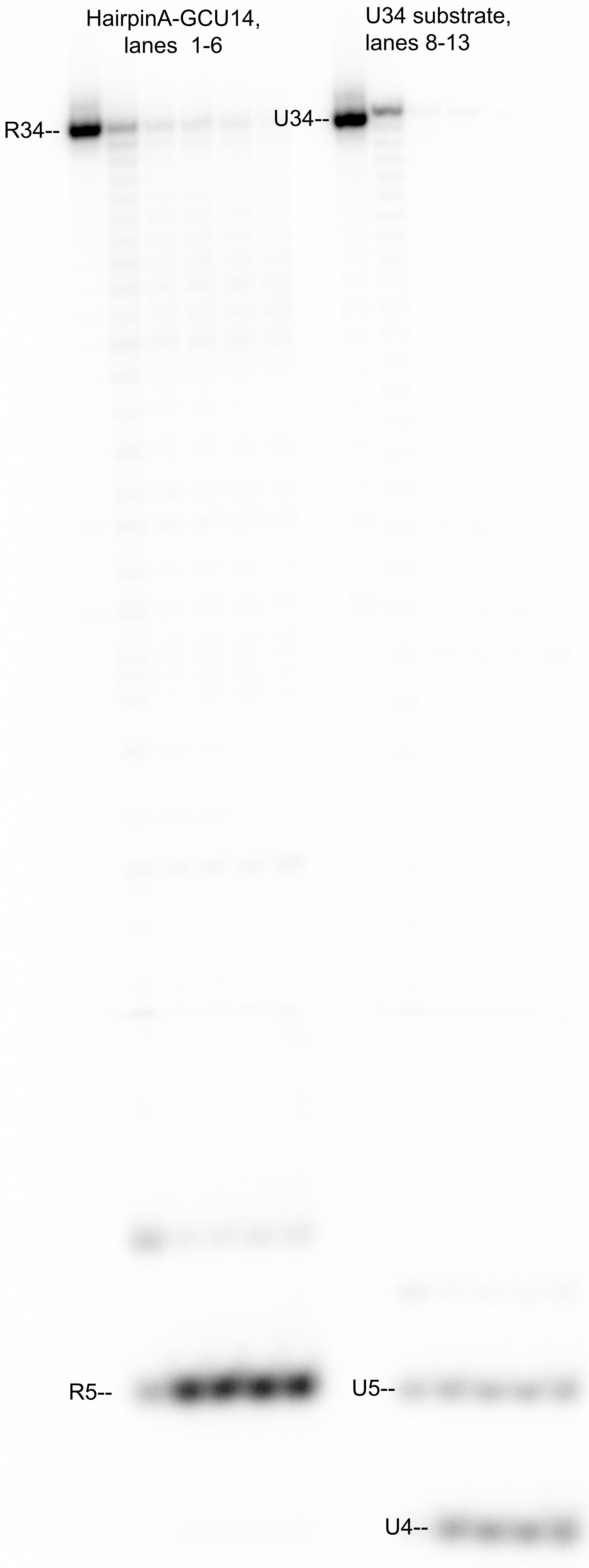
HairpinA-GCU14, lanes 1-6
U34 substrate, lanes 8-13
R34--
U34--
R5--
U5--
U4--
