# Supplementary material for: A shape-shifting nuclease unravels structured RNA
Source: Nat Struct Mol Biol. 2023 Feb 23;30(3):339–47. doi: 10.1038/s41594-023-00923-x (PMC10023572; doi:10.1038/s41594-023-00923-x)

ED Fig 8a, 7SL-HairpinF-U16, lanes 1-10

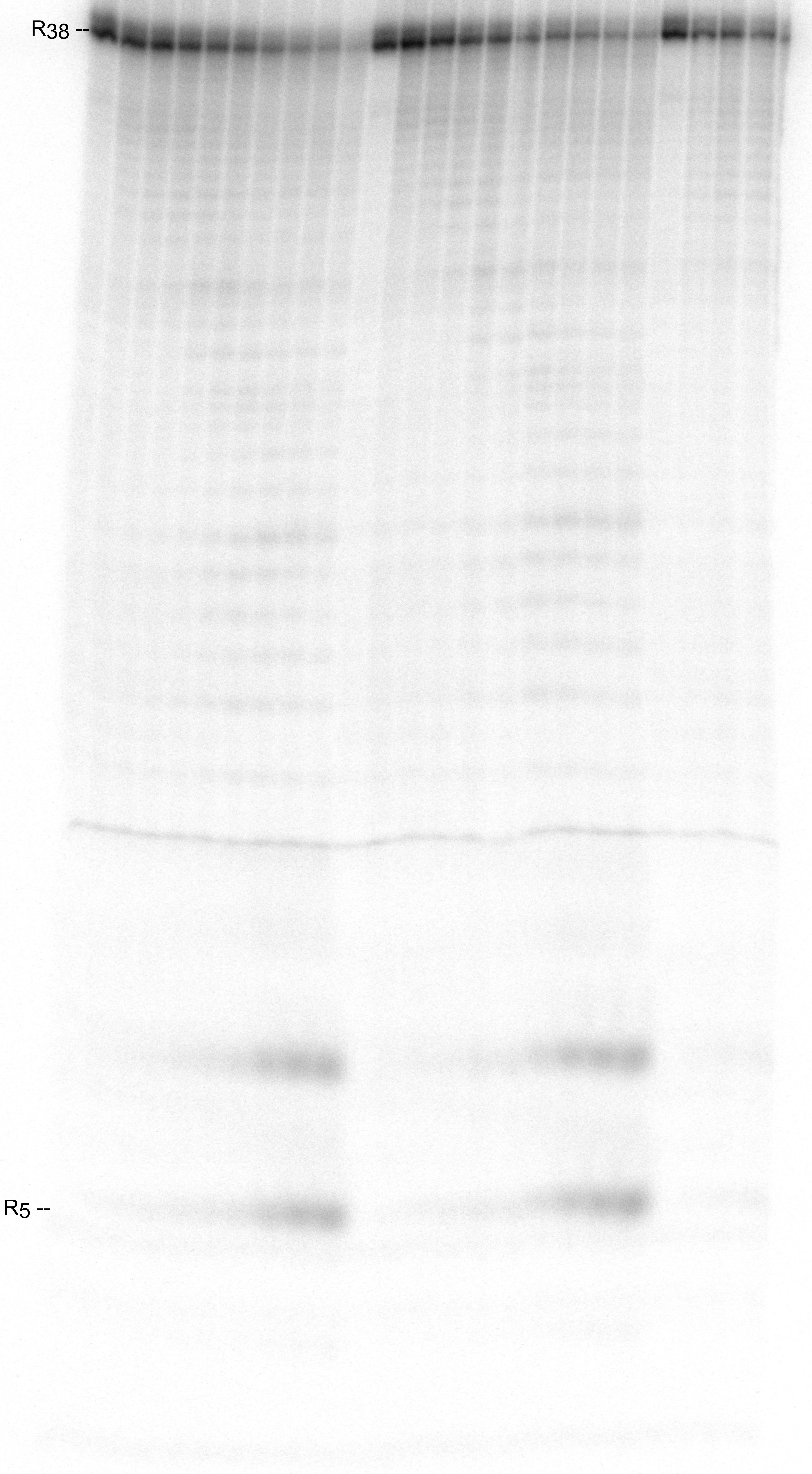

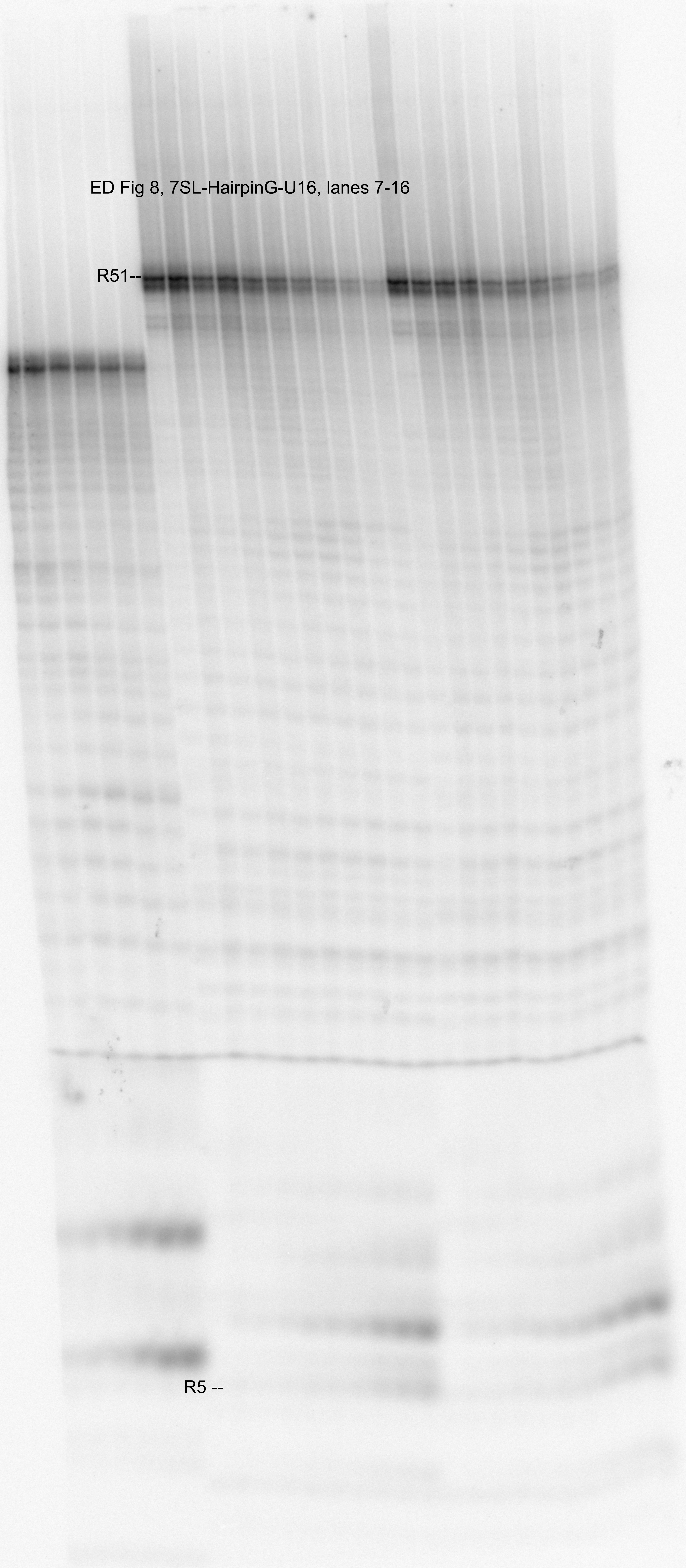
ED Fig 8, 7SL-HairpinG-U16, lanes 7-16
R51--
R5 --

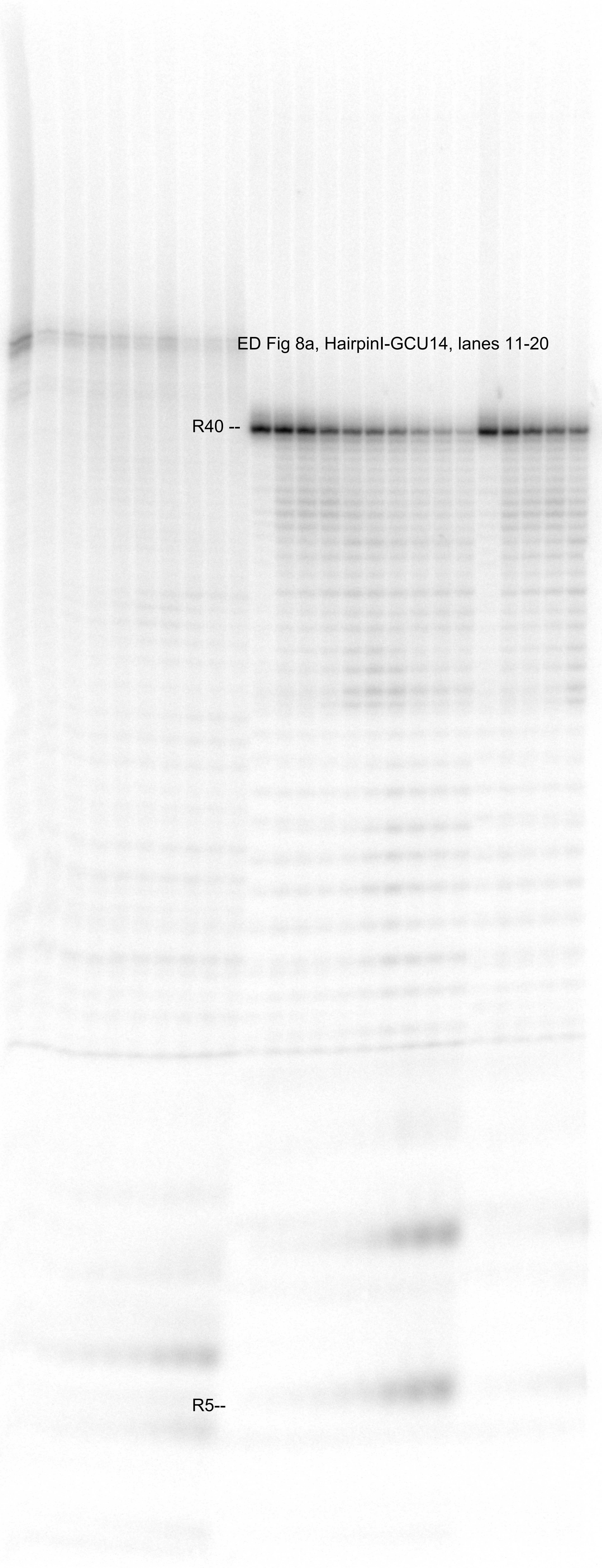

ED Fig 8a, HairpinI-GCU14, lanes 11-20
R40 --
R5--

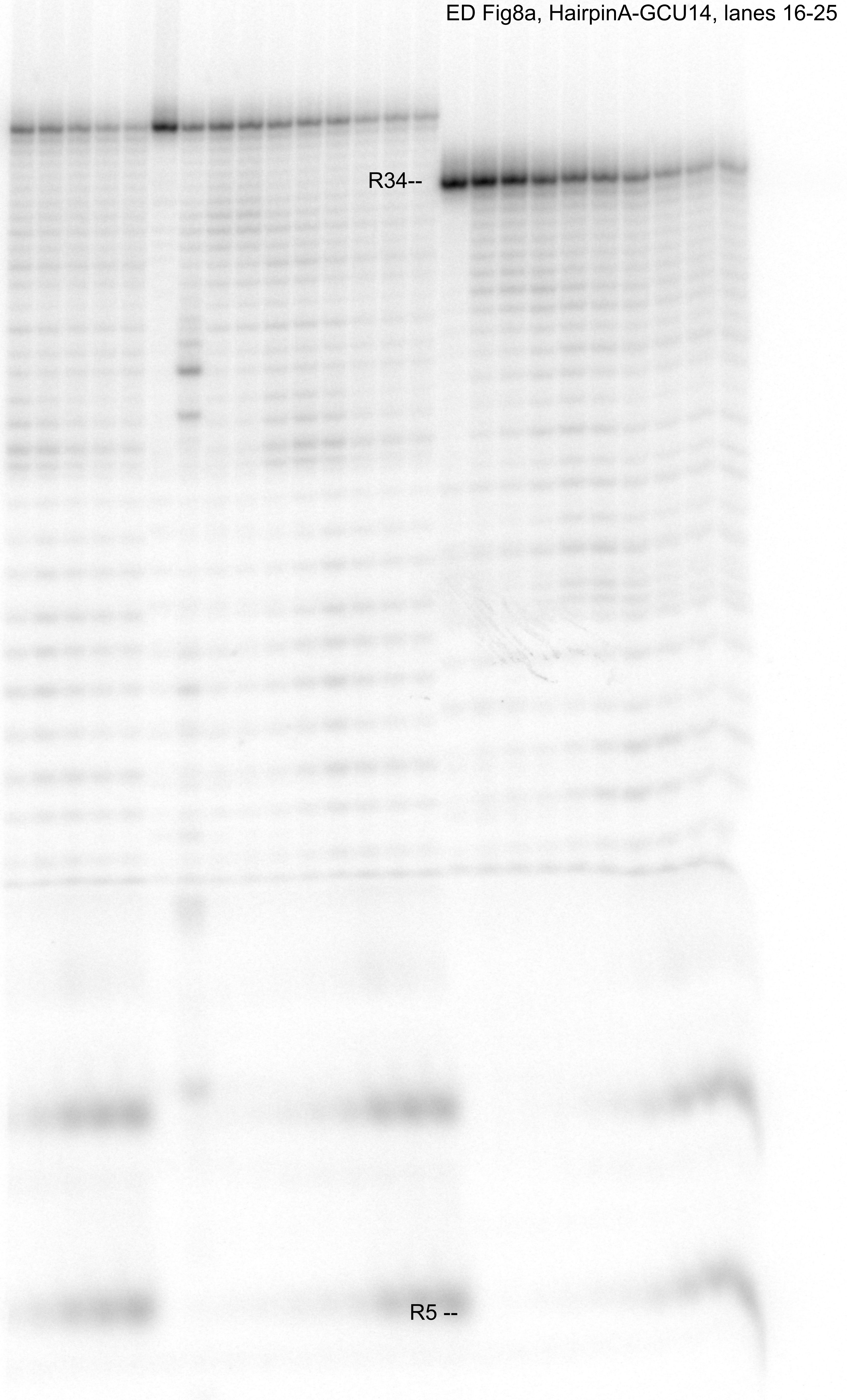
ED Fig8a, HairpinA-GCU14, lanes 16-25
R34--
R5 --

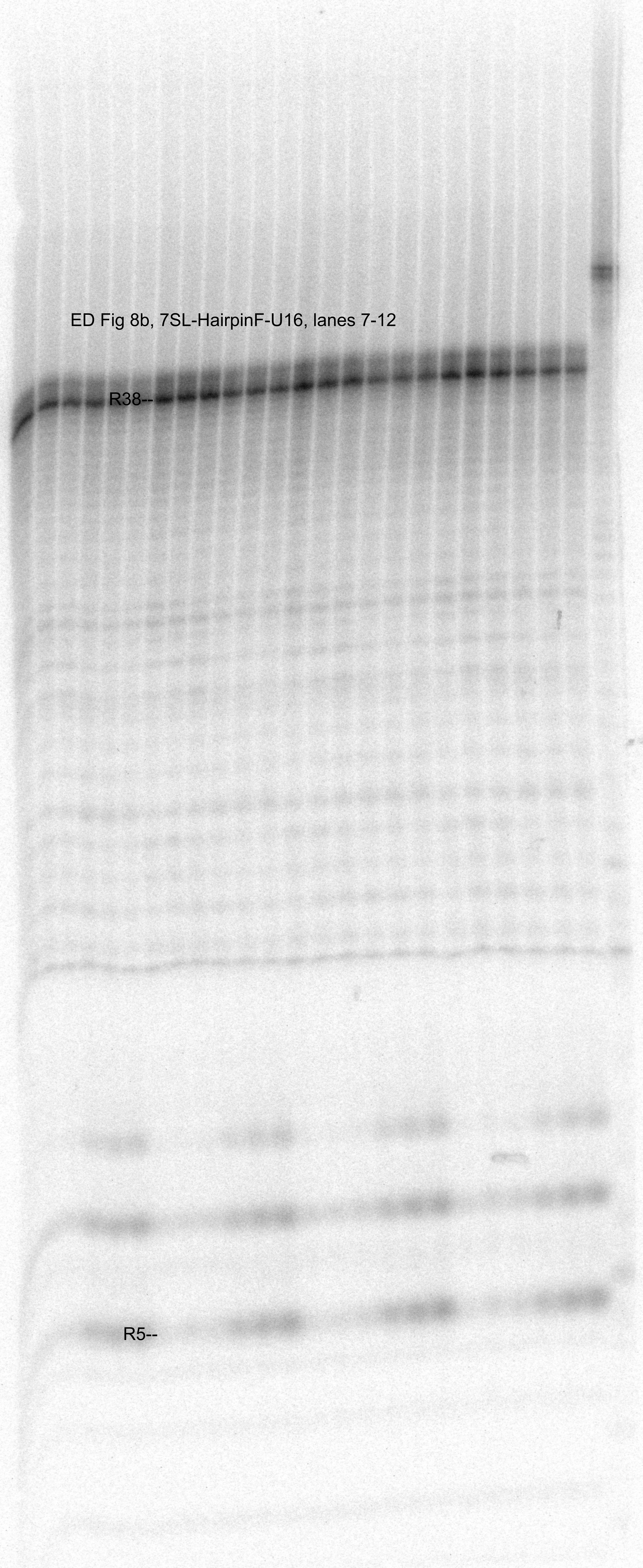
ED Fig 8b, 7SL-HairpinF-U16, lanes 7-12
R38--
R5--

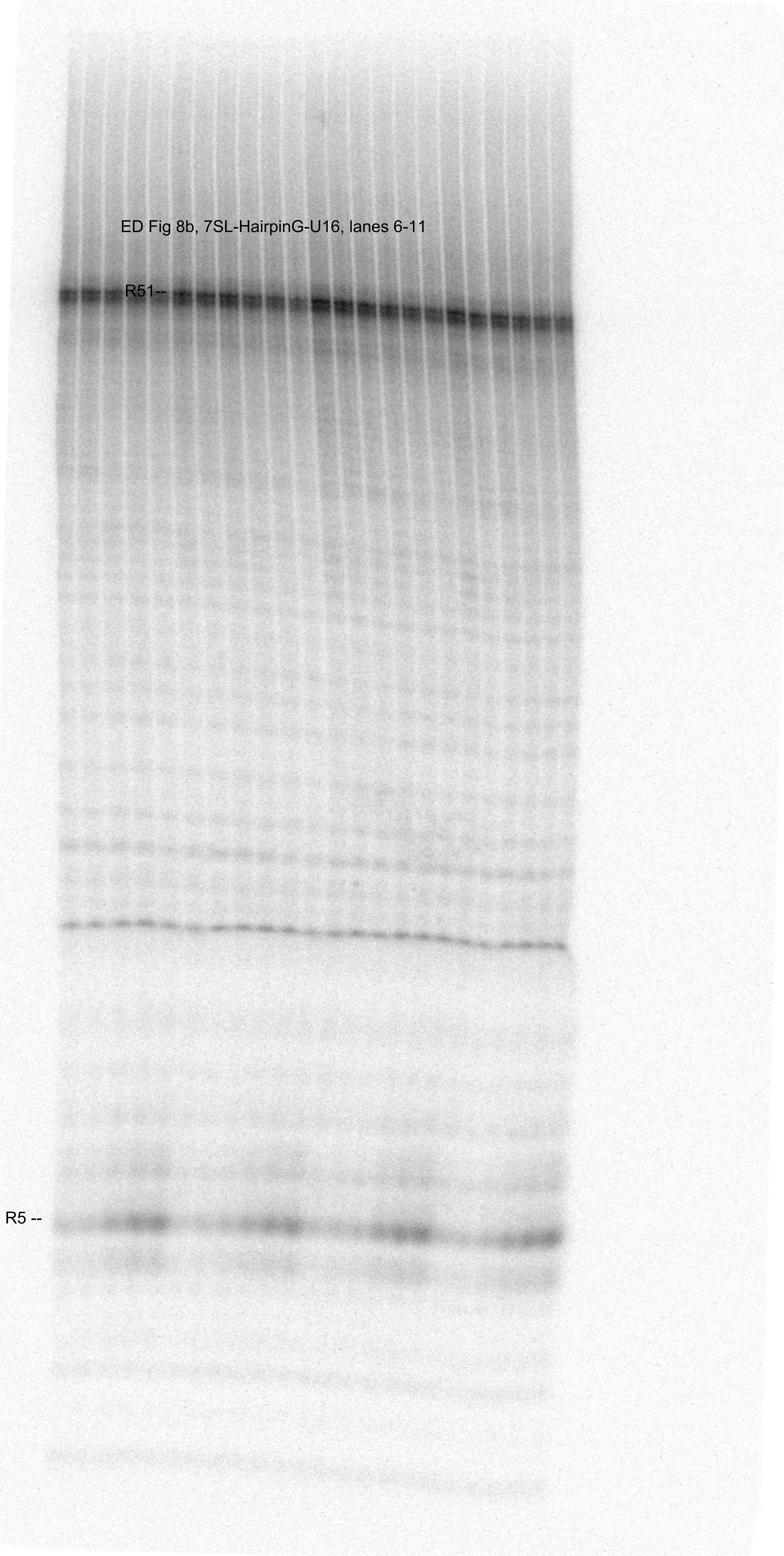
ED Fig 8b, 7SL-HairpinG-U16, lanes 6-11
R51--
R5 --

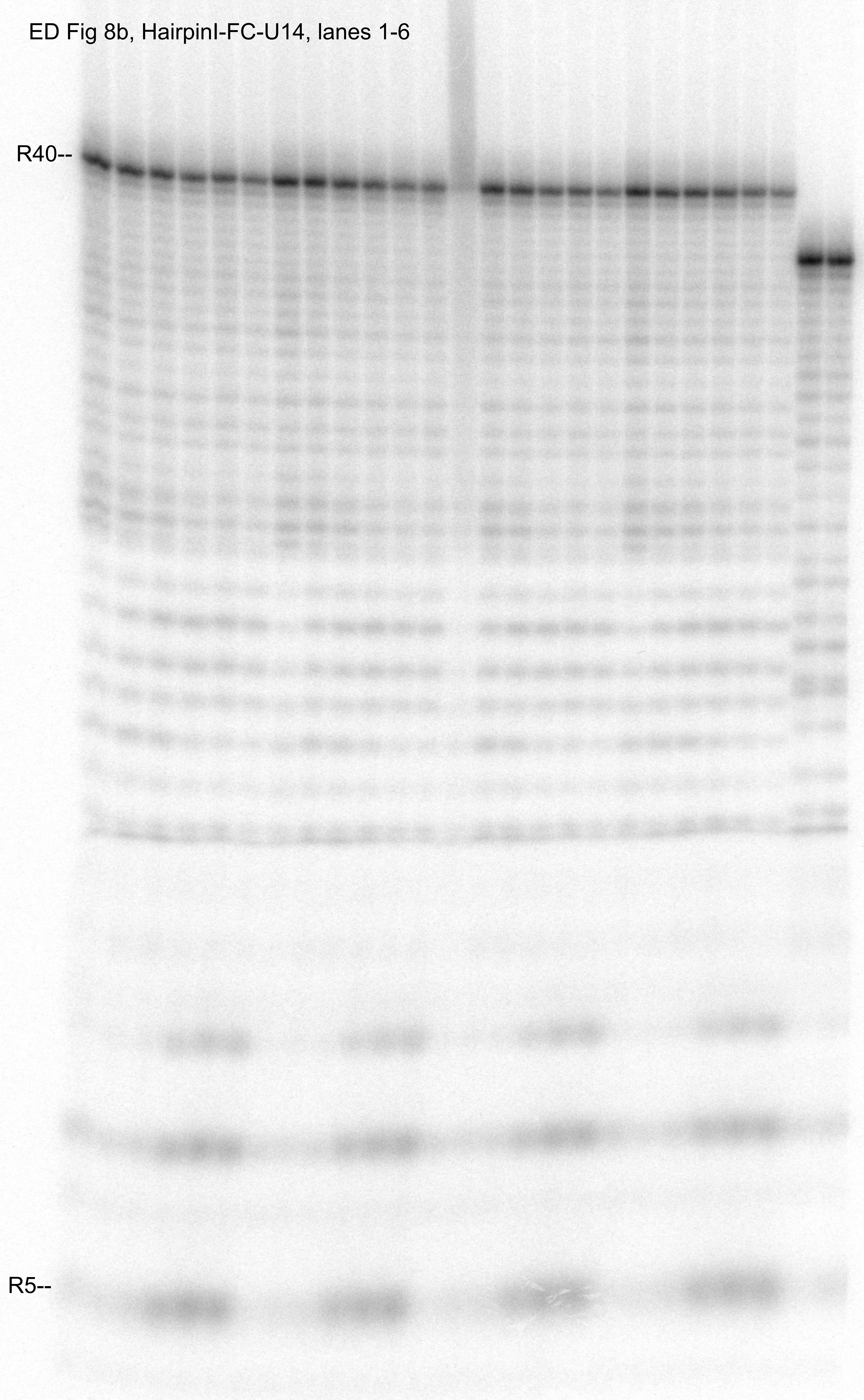
ED Fig 8b, HairpinI-FC-U14, lanes 1-6
R40--
R5--

ED Fig 8b, HairpinA-GCU14, lanes 5-10

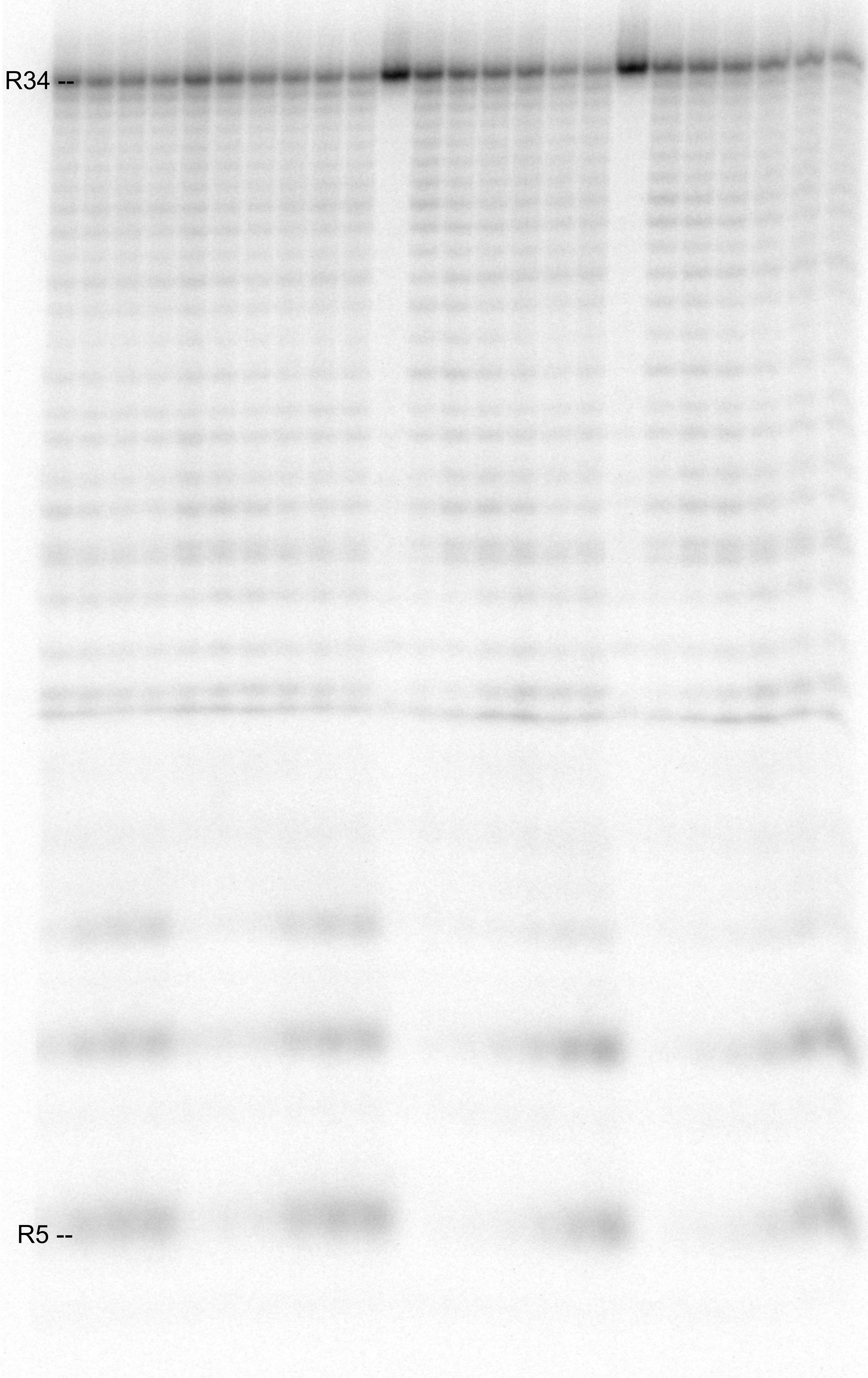

Supplement: Source Data Extended Data Fig. 8 — Source data (gels) for calculated kinetic parameters. [file 41594_2023_923_MOESM13_ESM.pdf]
