# Supplementary material for: A shape-shifting nuclease unravels structured RNA
Source: Nat Struct Mol Biol. 2023 Feb 23;30(3):339–47. doi: 10.1038/s41594-023-00923-x (PMC10023572; doi:10.1038/s41594-023-00923-x)
Supplement: Source Data Extended Data Fig. 10 — Source data (gels) for calculated kinetic parameters. [file 41594_2023_923_MOESM16_ESM.pdf]

ED Fig 10g, HairpinA-GCU14, lanes 7-16

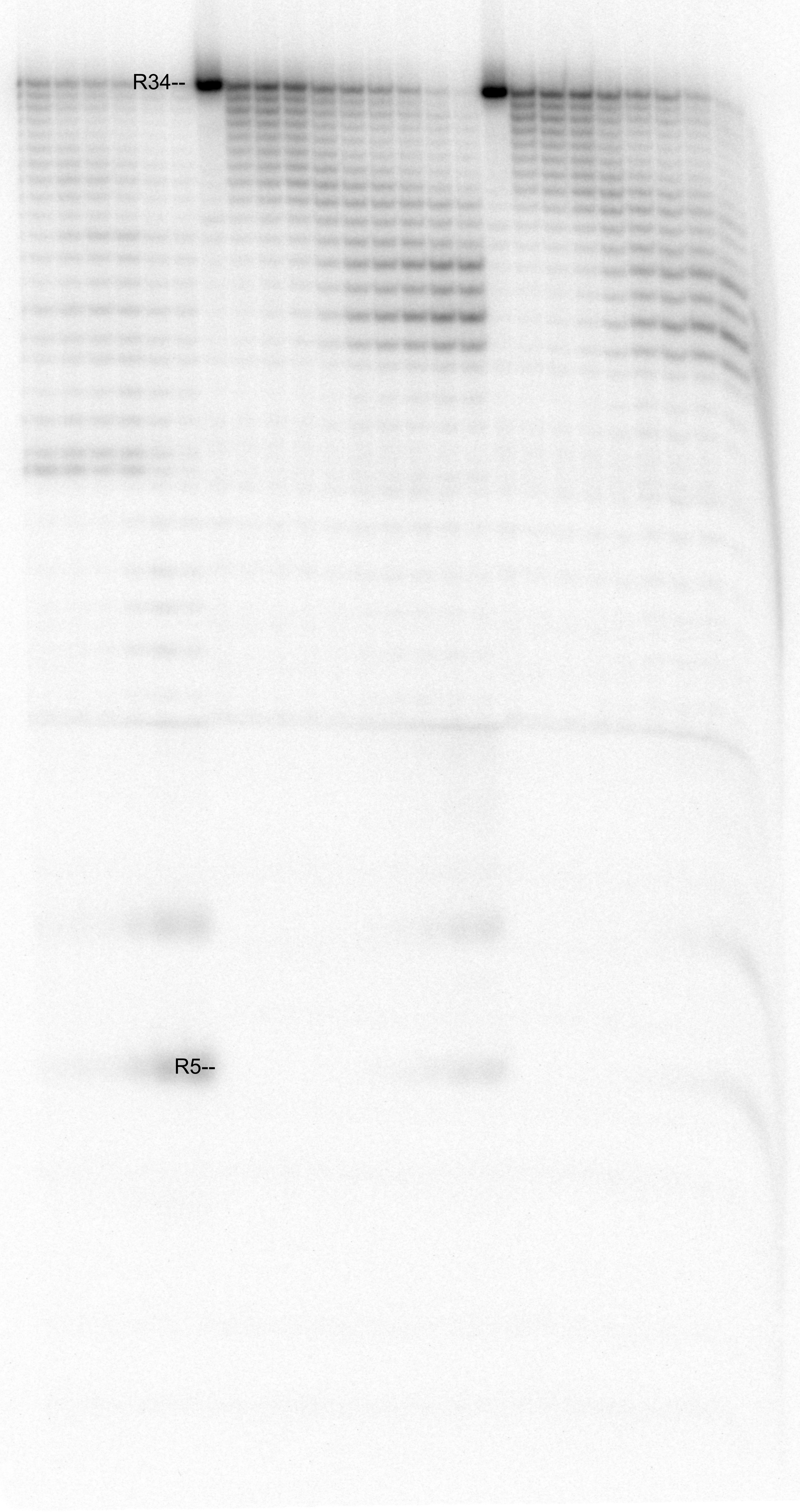

ED Fig 10g, U34 substrate, lanes 6-16

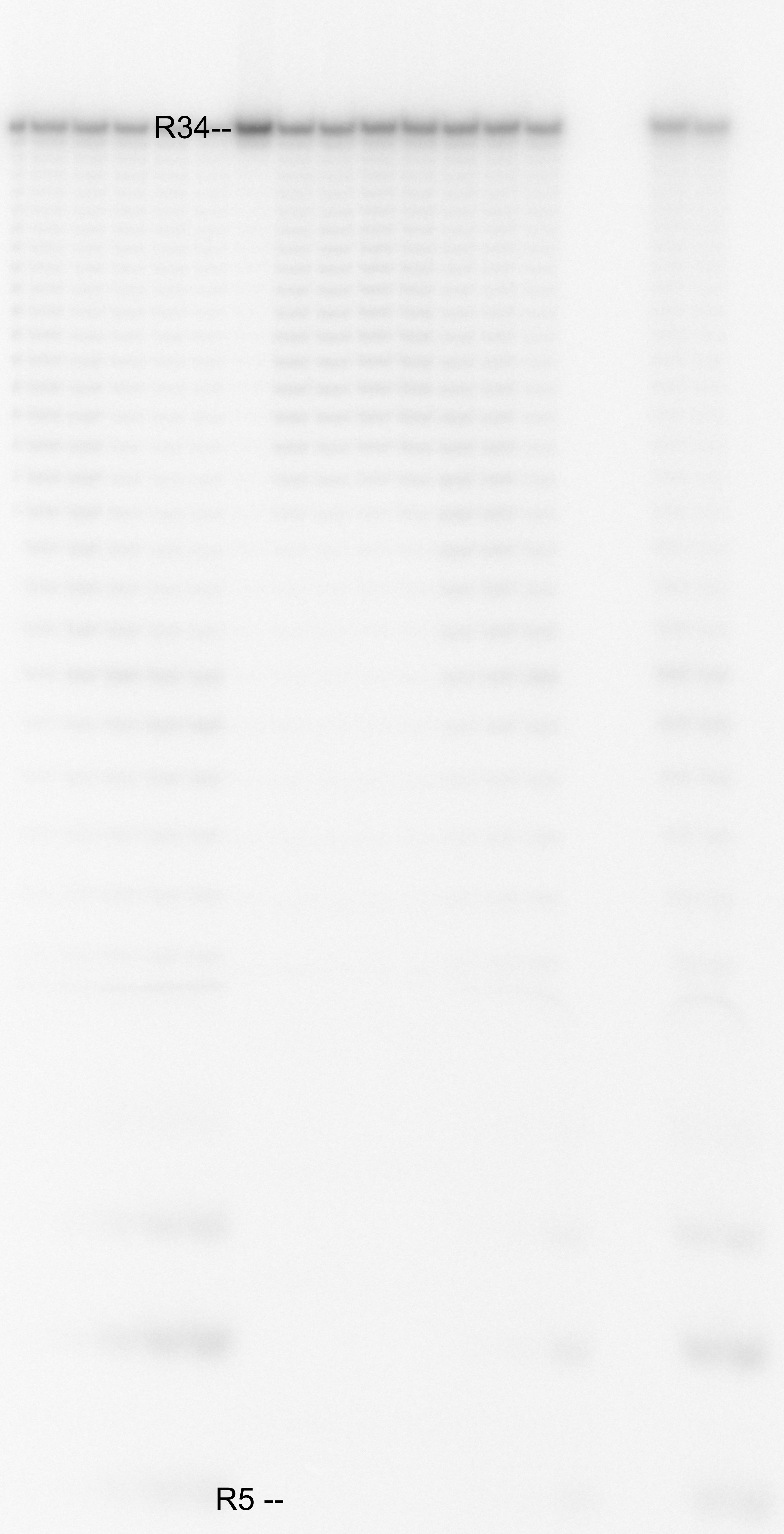

ED Fig 10k, d1H, lanes 11-16

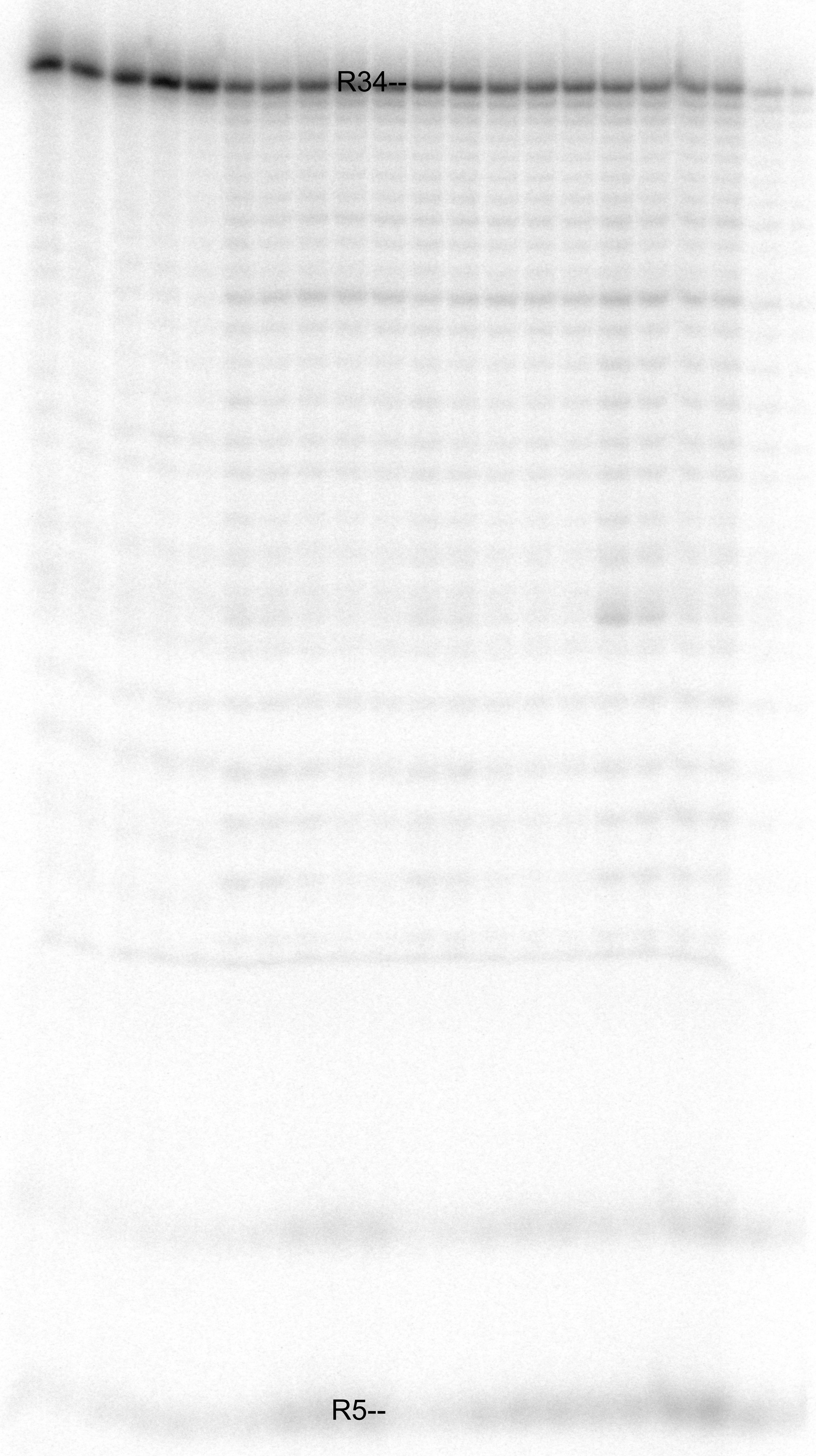

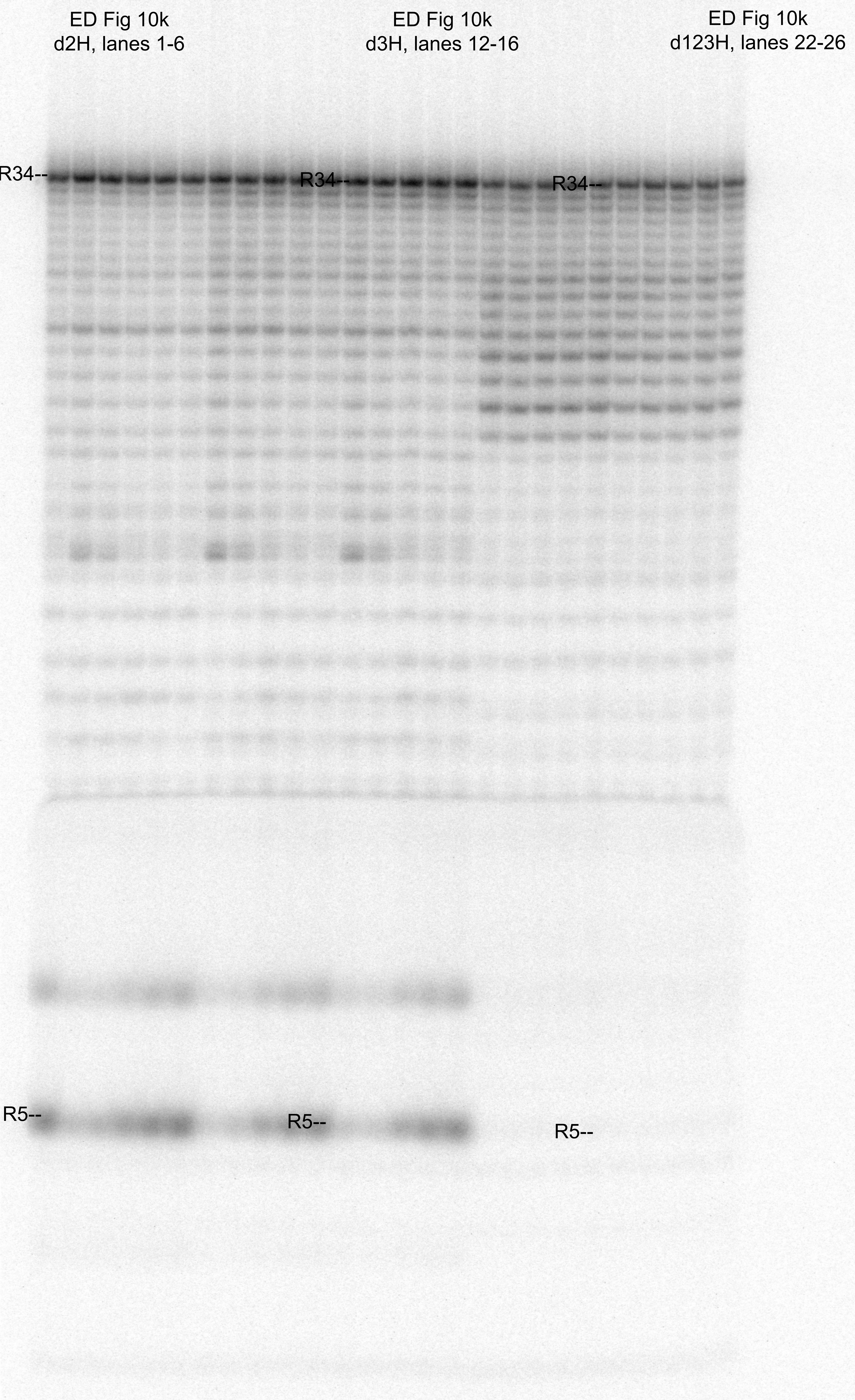
ED Fig 10k
d2H, lanes 1-6
ED Fig 10k
d3H, lanes 12-16
ED Fig 10k
d123H, lanes 22-26
R34--
R34--
R34--
R5--
R5--
R5--
